# Translation‐coupled mRNA quality control mechanisms

**DOI:** 10.15252/embj.2023114378

**Published:** 2023-08-22

**Authors:** Laura Monaghan, Dasa Longman, Javier F Cáceres

**Affiliations:** ^1^ MRC Human Genetics Unit, Institute of Genetics and Cancer University of Edinburgh Edinburgh UK

**Keywords:** RNA quality control, No‐go mRNA decay, Non‐stop mRNA decay, Nonsense‐mediated mRNA decay, UPF1, Chromatin, Transcription & Genomics, RNA Biology

## Abstract

mRNA surveillance pathways are essential for accurate gene expression and to maintain translation homeostasis, ensuring the production of fully functional proteins. Future insights into mRNA quality control pathways will enable us to understand how cellular mRNA levels are controlled, how defective or unwanted mRNAs can be eliminated, and how dysregulation of these can contribute to human disease. Here we review translation‐coupled mRNA quality control mechanisms, including the non‐stop and no‐go mRNA decay pathways, describing their mechanisms, shared trans‐acting factors, and differences. We also describe advances in our understanding of the nonsense‐mediated mRNA decay (NMD) pathway, highlighting recent mechanistic findings, the discovery of novel factors, as well as the role of NMD in cellular physiology and its impact on human disease.

## Graphical abstract

This review discusses mRNA quality control mechanisms including non‐stop and no‐go mRNA decay pathways and the nonsense‐mediated mRNA decay pathway.

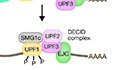

## Introduction

**Figure 1:**
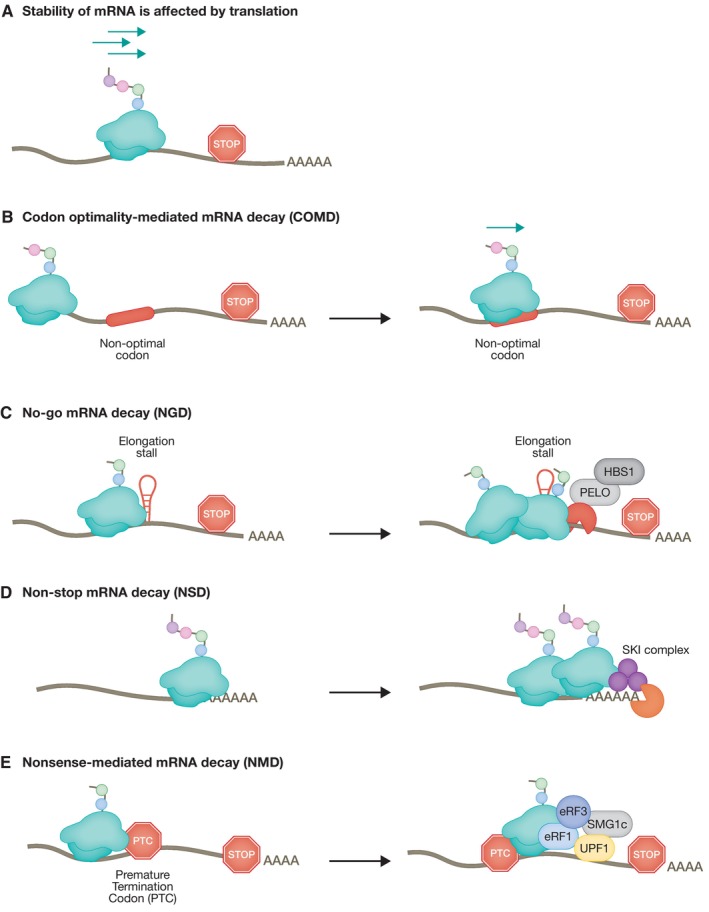
Translation‐coupled mRNA quality control mechanisms (A) Stability of mRNAs is affected by translation. Several layers of regulation monitor the efficiency of mRNA translation, including the translation rate, amino acid composition, and mRNA secondary structures. (B) The mRNA translation rate is slowed down when the ribosome encounters sub‐optimal codons leading to a decrease in mRNA stability. (C) No‐go mRNA decay (NGD) is triggered by the presence of mRNA secondary structures leading to ribosome stalling. (D) The absence of a stop codon results in slowing of the ribosome reading through the poly (A) tail triggering the non‐stop mRNA decay (NSD) pathway. (E) Recognition of a PTC sets in motion a cascade of event involving the UPF family of proteins, resulting in mRNA degradation by the nonsense‐mediated mRNA decay (NMD) pathway.

Translation‐coupled RNA quality control mechanisms sense ribosome stalling, or premature translation stops and elicit mRNA degradation and ribosome recycling. In eukaryotic cells, there is an intricate relationship between mRNA turnover and active translation (Fig 1A). This was recently confirmed in mammalian cells in culture, with the use of single‐molecule imaging approaches that revealed translation‐dependent destabilization of mRNA (Dave *et al*, 2023). In addition, the presence of a series of non‐optimal codons can negatively influence protein production by decreasing ribosome translocation rates leading to ribosome collisions that have the potential to trigger RNA quality control pathways and lead to mRNA decay (Hanson & Coller, 2018; Wu & Bazzini, 2023). This process, known as codon optimality‐mediated mRNA decay (COMD), allows the cell to distinguish between variation in normal translation speeds and terminal ribosome stalling, which triggers alternative RNA quality control pathways (Fig 1B) (Wu *et al*, 2019; D’Orazio & Green, 2021).

Two major pathways of co‐translational mRNA surveillance, no‐go mRNA decay (NGD) and non‐stop mRNA decay (NSD), sense aberrant translation by monitoring the stalling of ribosomes and/or ribosomes that fail to encounter a stop codon, respectively (Fig 1C and D). A common feature of both NGD and NSD is that upon sensing defective ribosome translocation, these pathways activate mechanisms leading to mRNA and nascent peptide degradation, and ribosome recycling (Brandman & Hegde, 2016; Powers *et al*, 2020). NGD and NSD use similar factors, but differ mainly in their substrate mRNAs and initial triggering mechanism (Simms *et al*, 2016). In both pathways, ribosome stalling is followed by recruitment of nucleases leading to degradation of mRNA (Powers *et al*, 2020). By contrast, the nonsense‐mediated mRNA decay (NMD) pathway senses inappropriate translation termination (Kervestin & Jacobson, 2012; Hug *et al*, 2016) (Fig 1E). NMD eliminates mRNAs that harbor premature termination codons (PTCs), thus, preventing the synthesis of truncated proteins. Significantly, NMD has a more global role in post‐transcriptional regulation of gene expression and also regulates the stability of many cellular non‐mutated transcripts, which do not harbor PTCs. These non‐canonical functions of the NMD pathway, which do not represent RNA quality control per se, are important for the regulation of many cellular pathways, including differentiation, neurogenesis, synaptic control, as well as the response to viral infections and stress (Jaffrey & Wilkinson, 2018; Kurosaki *et al*, 2019).

In this review article, we will focus primarily on three translation‐coupled RNA quality control mechanisms: NGD, NSD, and NMD. We will review similarities and differences among these pathways and cover recent advances related to their mechanisms, targets, and trans‐acting factors that ensure a tight control of aberrant RNAs that fail to be properly translated.

## No‐go mRNA decay

**Figure 2:**
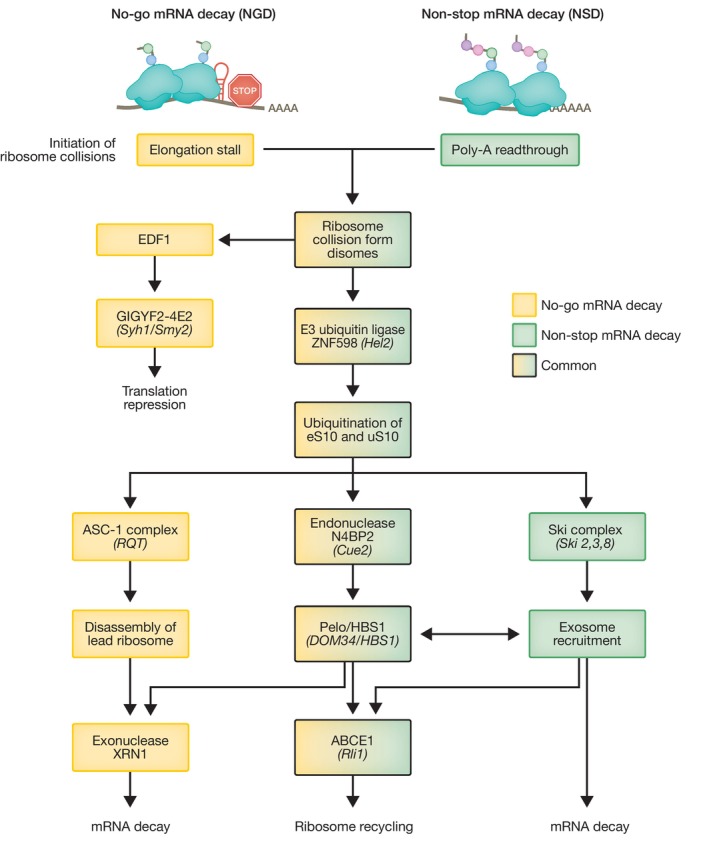
Steps and factors involved in no‐go and non‐stop mRNA decay Flow chart depicting steps and trans‐acting factors shared or unique to these two RNA quality control pathways from initial ribosome collision, ubiquitination and endonucleolytic cleavage to ultimate mRNA degradation and ribosome recycling. *S. cerevisiae* homologs are indicated in brackets.

Initially discovered in *S. cerevisiae*, the no‐go mRNA decay (NGD) pathway is triggered when ribosomes move slowly or stall during translation elongation, leading to ribosome collisions with trailing ribosomes, forming disomes (Doma & Parker, 2006; Harigaya & Parker, 2010). Ribosome stalling can be triggered by a number of stimuli, such as the presence of stable stem loops, pseudoknots, GC‐rich sequences, or damaged RNA bases (Shoemaker & Green, 2012). Persistent collisions trigger a two‐pronged cellular response that aims to inhibit translation re‐initiation on the problematic mRNA, and simultaneously remove faulty mRNAs and nascent peptides, and recycle the ribosomes (Fig 2).

Initially, translational repression is mediated by EDF1, which binds to collided ribosomes and recruits the translational repressor complex GIGYF2‐4E2 (Syh1‐Smy2 in *S. cerevisiae*) (Hickey *et al*, 2020; Sinha *et al*, 2020; Veltri *et al*, 2022). Upon ribosome collision, leading and trailing ribosomes form a ‘rotated’ interface of 40S subunits recognized by the E3 ubiquitin ligase ZNF598 (Hel2 in yeast) that ubiquitinates ribosomal proteins eS10 and uS10 and acts independently of the GIGYF2‐4E2 branch (Juszkiewicz *et al*, 2018; Ikeuchi *et al*, 2019) to initiate a degradation pathway. Ubiquitination of the 40S subunit is critical for the recruitment of an endonuclease, which cleaves mRNAs in the middle of a stalled disome, exposing fragmented mRNA to further degradation by XRN1 and potentially the exosome. A genetic screen in yeast recently identified the endonuclease Cue2 as a component of the NGD pathway (D’Orazio *et al*, 2019). Cue2 is a low‐abundance protein that cleaves mRNA at the A site of the rotated collided ribosome in a Hel2‐dependent manner and has homologues in *C. elegans* (NONU1) and in mammalian cells (N4BP2; D’Orazio *et al*, 2019; Glover *et al*, 2020; Monem *et al*, 2023). As a consequence of mRNA cleavage, stalled ribosomes appear near the 3′ end of mRNA, which favors the binding of an evolutionarily conserved complex formed by PELOTA (PELO) and its GTPase cofactor HBS1 (Dom34/Hbs1 in yeast; Doma & Parker, 2006; Pisareva *et al*, 2011). Despite structural similarities with release factors eRF1/eRF3, instead of detecting a stop codon, the PELO/HBS1 complex preferentially binds an empty ribosomal A site. This induces ribosome splitting into 40S bound to the mRNA, and 60S with the attached nascent peptide, and is mediated by the ribosome recycling factor ABCE1 (Rli1 in yeast; Shoemaker *et al*, 2010). The nascent peptide is subsequently targeted for Listerin‐mediated ubiquitination and degraded by the proteasome as part of the ribosome quality control (RQC) pathway (Shao *et al*, 2013). However, despite widespread conservation of the Cue2‐Dom34/Hbs1 pathway, recent evidence suggests that this represents a secondary decay method in NGD and may only be required when the capacity of the XRN1‐mediated degradation pathway is saturated (Juszkiewicz *et al*, 2020). In the majority of mRNAs with ribosome stalls within the open reading frame (ORF), Hel2‐mediated 40S ubiquitination of disomes recruits the ribosome quality control trigger (RQT) complex containing the RNA helicase Slh1 in yeast, with the homologous complex ASC‐1 performing the same role in mammals (Matsuo *et al*, 2017). Upon 40S ubiquitination of collided ribosomes, the RNA helicase ASCC3 (part of the ASC‐1 complex) selectively disassembles the lead ribosome in an ATP‐dependent manner, in a process not requiring PELO/HBS1 activity (Juszkiewicz *et al*, 2020; Matsuo *et al*, 2020). After removing the roadblock of the lead stalled ribosome, normal elongation can resume, or in case of persistent stalls, subsequent ribosome collisions induce further quality control and mRNA is subjected to XRN1‐mediated degradation, likely with a contribution of the PELO/HBS1 branch of NGD.

## Non‐stop mRNA decay

The non‐stop mRNA decay pathway (NSD) degrades mRNAs that lack a stop codon, ensuring the degradation of mRNAs that will fail to be properly translated (Frischmeyer *et al*, 2002; van Hoof *et al*, 2002). It can be initiated by a number of aberrant translation events which promote readthrough into the poly(A) tail resulting from lack of an in‐frame stop codon or polyadenylation within the ORF. Poly(A) translation within the coding region slows down when the nascent poly(lysine) peptide (encoded by poly(A)) interferes with the ribosomal exit tunnel. This attenuation of translation elongation allows stretches of poly(A) to adopt a conformation that impedes further mRNA decoding at the ribosome A site resulting in stalling of the translating ribosome (Ito‐Harashima *et al*, 2007; Chandrasekaran *et al*, 2019; Tesina *et al*, 2020). Interestingly, hindered elongation can induce ribosome sliding on poly(A) sequences causing frameshifting that may lead to an out‐of‐frame PTC and subsequent triggering of NMD‐mediated mRNA degradation (Koutmou *et al*, 2015). Importantly, elongation stalling causes collisions with trailing ribosomes and the resulting disomes are recognized and ubiquitinated by the ZNF598 E3 ubiquitin ligase with subsequent Cue2‐mediated endonucleolytic cleavage, steps that are also common to NGD, as described above (Juszkiewicz & Hegde, 2017; Powers *et al*, 2020).

In yeast, 80S ribosomes stalled at the 3′end of an mRNA lacking a stop codon are bound by the SKI complex, leading to recruitment of the exosome by the exosome‐associated factor SKI7, resulting in degradation of the aberrant mRNA in a 3′ to 5′ direction. SKI7 binds the empty A site of the stalled ribosome at the 3′ end of mRNA and directs mRNA degradation through an eRF3‐like domain (Saito *et al*, 2013). This process also requires the RNA helicase SKI2 (SKIV2L in mammals) to guide RNA molecules to the exosome complex, triggering mRNA degradation upon ribosome stalling on A‐rich sequences (Tuck *et al*, 2020). In mammalian cells where SKI7 is not present, bridging the SKI complex to the exosome is carried out by HBS1, its closest homologue. This triggers the interaction with Dom34/Pelota‐Hbs1 complex shared with NGD and acts to dissociate the stalled ribosome with the help of ribosome recycling factors Rli1 (yeast)/ABCE1 (mammals; Tsuboi *et al*, 2012; Saito *et al*, 2013).

Fundamentally, NGD and NSD are parallel mRNA quality control pathways that share many similarities, such as ubiquitination of crucial ribosome sites, Cue2‐mediated endonucleolytic cleavage, activation of mRNA decay, and ribosome recycling; however, they have some key differences (Fig 2). The NSD helicase SKI2 is highly related to the NGD helicase Slh1 in yeast/ASCC3 in mammals; however, the Ski complex directly recruits the exosome, thus directly linking mRNA degradation and ribosome recycling. Conversely, in NGD, the Slh1/ASCC3 complex removes stalled ribosomes first, resulting in mRNA degradation mediated by XRN1. NSD has been implicated in the development of several human diseases, highlighting the physiological importance of this RNA quality control pathway. For example, non‐stop mutations in the Dysferlin gene (*DYSF*) lead to degradation of its mRNA and a subsequent reduction in *Dysferlin* expression, contributing to the progression of muscular dystrophy (Cacciottolo *et al*, 2011). Insufficient NSD activity can also lead to disease, such as mutations which eliminate the stop codon in the skeletal muscle alpha actin (*ACTA1*) gene. Here, incomplete NSD fails to remove mRNAs encoding 47 additional amino acids that are translated within the 3′UTR, leading to large protein aggregates that are responsible for the development of severe skeletal myopathy (Wallefeld *et al*, 2006).

## Nonsense‐mediated mRNA decay

NMD is an RNA quality control mechanism that targets mutated mRNAs harboring PTCs for degradation, but also regulates the stability of many cellular transcripts. As such, NMD modulates the phenotypic outcome of genetic disorders caused by frameshift or nonsense mutations that generate PTCs (Bhuvanagiri *et al*, 2010; Karousis & Mühlemann, 2022). In contrast to NGD and NSD, which are triggered by the collision of elongating ribosomes, NMD initiates mRNA degradation in response to faulty translation termination events. Similar to NGD and NSD, NMD degrades mRNAs co‐translationally, leading to production of truncated nascent peptides. In the case of NGD and NSD, these potentially toxic peptides are rapidly degraded by the RQC pathway. By contrast, peptides produced from PTC‐containing transcripts are targeted by an ubiquitin proteasome system, which specifically targets peptides remaining tethered to the ribosome following decay of a nonsense mRNA (Inglis *et al*, 2023).

The function of NMD in preventing the accumulation of truncated proteins can have a positive or negative cellular effect, depending on whether the truncated protein has a deleterious dominant negative function, or whether it retains at least partial function, elimination of which by NMD could be detrimental. For example, inhibition of NMD by antisense oligonucleotides (ASOs) targeting the W1282X mutation in the CFTR gene increases production of a partially functional protein that enhances the CFTR‐mediated chloride current in human bronchial cells (Kim *et al*, 2022). On the other hand, failure of NMD to degrade β‐globin transcripts harboring a PTC in the last exon leads to expression of truncated, dominant‐negative protein causing severe beta‐thalassemia (Hall & Thein, 1994). The effect of NMD must be carefully considered in the development of any therapeutic approach for diseases caused by PTC mutations. Importantly, NMD also has a role in many physiological processes, for example in the regulation of the stress response and as a modulator of neural development (Jaffrey & Wilkinson, 2018; Kurosaki *et al*, 2019).

In mammals, NMD is linked to the process of pre‐mRNA splicing via the exon junction complex (EJC), a multi‐subunit protein complex that is deposited 20‐24 nucleotides upstream of most exon‐exon junctions (Le Hir *et al*, 2000a, 2000b). The splicing factor CWC22 interacts with the core EJC factor eIF4AIII and links splicing with EJC deposition, leading to NMD activation (Alexandrov *et al*, 2012; Barbosa *et al*, 2012; Steckelberg *et al*, 2012). Mechanistically, the NMD response is coupled to mRNA translation since EJCs remain bound to mRNAs until they are displaced by the translation machinery. Initially, it was suggested that NMD activation occurs as a consequence of ribosome stalling at the termination codon (TC). However, recent data challenge this model and revealed that NMD activation in humans is not necessarily linked to stable stalling of ribosomes at TCs (Karousis *et al*, 2020). A ribosome terminating prematurely at a PTC located ≥ 50–55 nucleotides upstream of the final exon–exon junction will not remove an EJC (Nagy & Maquat, 1998; Thermann *et al*, 1998) and this initiates the NMD response (Metze *et al*, 2013; Kurosaki *et al*, 2019).

### Core NMD factors

**Figure 3:**
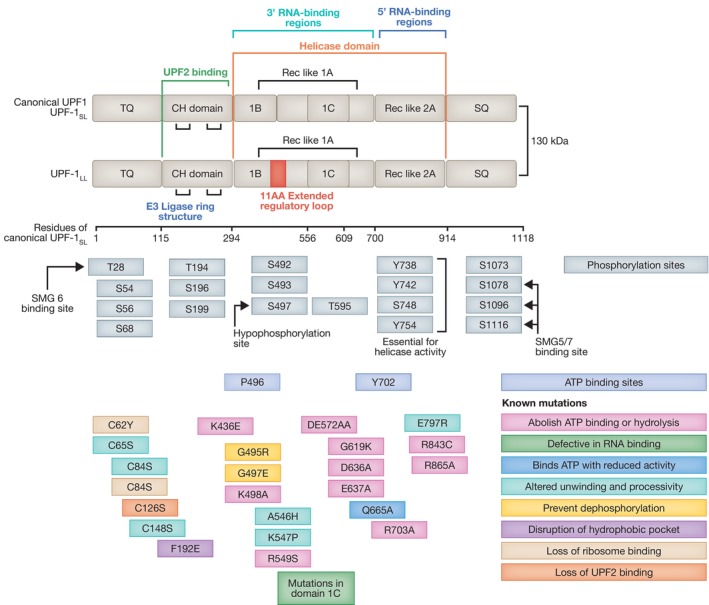
UPF1 structure, binding sites, and known mutations Diagram depicting the domain structure of the canonical UPF1_SL_ isoform, highlighting key residues for its phosphorylation and ATP binding capacity. An isoform generated via alternative splicing, UPF_LL_, includes an extra 11 amino acid extended regulatory region, while all other structural elements remain the same. Mutations and their effect on UPF1 function are indicated by the colored boxes. Some mutations in UPF1 have the capacity to eliminate NMD function, whilst some mutations sustain functional NMD. All residue numbers relate to the canonical UPF1_SL_ isoform.

A central player in the NMD pathway is the ATP‐dependent RNA helicase of the SF1 superfamily, Upstream Frameshift 1 (UPF1), which harbors an amino‐terminal cysteine‐and histidine‐rich (CH) domain and a carboxy‐terminal RNA helicase domain (Fig 3; Kim & Maquat, 2019).

**Figure 4:**
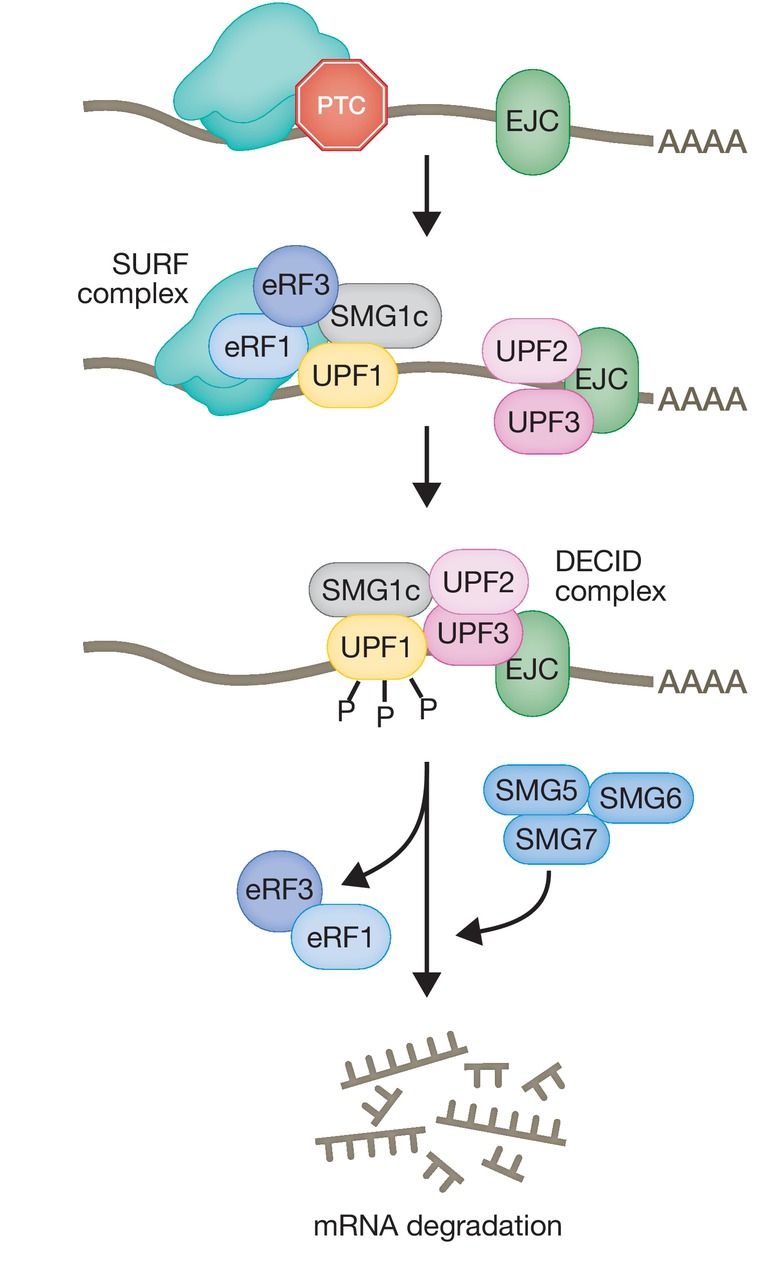
Mechanism of NMD activation Schematic depicting the widely accepted molecular events leading to the assembly of the surveillance complex (SURF), its transition to the decay‐inducing complex (DECID) leading to UPF1 phosphorylation and recruitment of SMG6 and/or SMG5/SMG7 that elicit mRNA degradation.

UPF1 is activated by phosphorylation carried out by the SMG1c complex, comprised of the phosphoinositide 3‐kinase (PI3K)‐like kinase, SMG1, and two additional subunits, SMG8 and SMG9, that negatively regulates its activity (Yamashita *et al*, 2001, 2009; Fernández *et al*, 2011; Langer *et al*, 2021). SMG1 phosphorylates UPF1 at multiple SQ and TQ motifs located in the amino‐ and carboxy‐terminal domains (Yamashita *et al*, 2001). Until recent years it was widely accepted that initially, the association of UPF1 with SMG1 and the eukaryotic release factors eRF1 and eRF3 form the surveillance complex (SURF) in the vicinity of the PTC (Kashima *et al*, 2006) (Fig 4). Subsequently, interaction of the SURF complex with UPF2 and UPF3B and an EJC downstream of the PTC leads to the assembly of a decay‐inducing complex (DECID), where UPF1 is phosphorylated and eRF1 and eRF3 are released (Kashima *et al*, 2006; Chamieh *et al*, 2008; López‐Perrote *et al*, 2016). In addition to this, a central role for UPF3B in translation termination has been highlighted. A fully reconstituted *in vitro* translation system showed the predominance of the interaction of UPF3B with ribosome release factors, to delay translation termination and dissociate post‐termination ribosomal complexes that are devoid of the nascent peptide. UPF1 was shown to interact transiently with the termination factors and UPF3B to initiate the subsequent mRNA decay (Neu‐Yilik *et al*, 2017). Despite the lack of mammalian *in vivo* data, a recent *in vivo* study in yeast re‐established the central role of the UPF1:80S interaction for translation termination and NMD initiation (Ganesan *et al*, 2022).

Under normal conditions UPF1 has low basal helicase activity; however, upon recognition of a PTC and subsequent binding of UPF1 to UPF2, this activity is greatly increased. Alongside this, a large conformational change of the CH inhibitory domain modifies the RNA‐binding properties and the catalytic activity of UPF1, causing a switch from an RNA‐clamping mode to an RNA‐unwinding mode (Chakrabarti *et al*, 2011). The active UPF1 helicase functions as an RNPase translocating along the mRNA with a 5′ to 3′ polarity, acting to resolve secondary structures, remove proteins from mRNA, and provide access to nucleases (Franks *et al*, 2010; Fiorini *et al*, 2015). Importantly, NMD can also be elicited on mRNAs that do not have a downstream EJC, although the mechanism of non‐EJC dependent NMD is less well defined (Bühler *et al*, 2006; He & Jacobson, 2015). Interestingly, alternative branches of the NMD pathway that act independently of UPF2, UPF3B, or the EJC have also been described (Gehring *et al*, 2005; Chan *et al*, 2007; Ivanov *et al*, 2008). In mammals, there are two highly related UPF3 paralogs, UPF3A and UPF3B (also called UPF3X due to its location in chromosome X). Recent studies have shown that UPF3A and UPF3B perform redundant functions and can activate NMD without EJC binding, suggesting that UPF3 paralogs play a more active role in NMD than simply bridging the EJC and the UPF complex. UPF1 almost exclusively associates with UPF3B and only minimally with UPF3A; however, when UPF3B is mutated or removed, the association of UPF1 with UPF3A is enhanced 4‐6 times, independent of RNA. Thus, UPF3A seems almost dispensable for NMD; however, it performs a compensatory role and can maintain NMD in the absence of UPF3B (Wallmeroth *et al*, 2022; Yi *et al*, 2022; Chen *et al*, 2023). In cells lacking both UPF3 paralogs, although NMD is not completely abrogated, its activity is reduced significantly (Bufton *et al*, 2022; Yi *et al*, 2022).

A current model postulates that UPF1 binding to mRNAs does not inevitably mark mRNAs for NMD‐mediated degradation. Use of CLIP (cross‐linking and immunoprecipitation) revealed that UPF1 binds target RNAs prior to mRNA translation. Once translating ribosomes are engaged, they displace UPF1 from coding sequences, leading to UPF1 enrichment at 3′UTRs (Hurt *et al*, 2013; Zünd *et al*, 2013). By contrast, binding of phosphorylated UPF1 (P‐UPF1) marks mRNAs for NMD‐mediated degradation, since P‐UPF1 is enriched on endogenous transcripts degraded by NMD, whereas unphosphorylated UPF1 is released from non‐targeted transcripts in an ATP‐dependent manner (Kurosaki *et al*, 2014; Lee *et al*, 2015). Interestingly, UPF1 mutants with substantially impaired processing and slower unwinding rates are still functional in NMD and still have the capacity to restore NMD functionality upon loss of WT UPF1 (Fig 3) (Chapman *et al*, 2022).

In addition to a rapidly increasing number of NMD regulators, it was recently shown that UPF1_LL_, an alternative mammalian‐specific isoform of the core NMD factor UPF1 (UPF1_SL_), harbors a regulatory loop that is 11‐residues longer and preferentially binds and down‐regulates a different subset of NMD targets through a reduction in the RNA dissociation potential (Fig 3) (Gowravaram *et al*, 2018; Fritz *et al*, 2022). Canonical NMD is mediated by UPF1_SL_ that represents >75% of the total UPF1 pool and is inhibited by moderate translational repression. By contrast, the UPF1_LL_ isoform triggers NMD in response to activation of the integrated stress response (ISR), and repression of translation, targeting novel mRNA, including stress response genes (Fritz *et al*, 2022). Interestingly, UPF1_LL_ requires translation termination events; however, due to its improved RNA‐binding capacity in the presence of ATP compared to UPF1_SL_, its sustained RNA interaction favors a reduced frequency of termination events in conditions of attenuated translation. Therefore, under conditions of moderate translation inhibition where NMD is inhibited, UPF1_LL_ activity is enhanced, changing the specificity of NMD in response to stress conditions (Fritz *et al*, 2022).

UPF1 phosphorylation represents a non‐reversible point in NMD progression during which the transcript is committed for degradation, leading to repression of further translation initiation, a key step in the NMD pathway (Isken *et al*, 2008). This phosphorylation event leads to the recruitment of additional NMD factors, namely the phospho‐binding proteins SMG6 and/or SMG5/SMG7, which function in two independent, yet overlapping pathways, and lead to further recruitment of nucleases to elicit mRNA degradation. The SMG6 endonuclease cleaves NMD targets in the vicinity of the PTC (Huntzinger *et al*, 2008; Eberle *et al*, 2009) and generates a 5′ cleavage product that is most likely degraded 3′ to 5′ by the exosome and/or DIS3L2 (DIS3‐like exonuclease 2; Kurosaki *et al*, 2018). The resulting 3′ cleavage product is cleared of protein components by UPF1 to provide access to the exoribonuclease XRN1 (Kurosaki *et al*, 2019). Alternatively, the heterodimer SMG5/SMG7 binds to P‐UPF1 and recruits the CCR4/NOT complex to promote deadenylation, leading to 3′ to 5′ decay (Loh *et al*, 2013) and decapping, resulting in XRN1‐catalyzed 5′ to 3′ degradation (Unterholzner & Izaurralde, 2004). Transcriptome profiling revealed that SMG6 and SMG7 act on essentially the same transcripts, suggesting extensive redundancy between the endo‐ and exonucleolytic decay pathways (Colombo *et al*, 2017). There is a tight regulation between these two RNA degrading pathways, since inactivation of the SMG5‐SMG7 pathway also abrogates the SMG6‐dependent mechanism, supporting a mechanism that involves UPF1 phosphorylation and SMG5‐SMG7 recruitment to access SMG6 activity (Boehm *et al*, 2021).

### Additional NMD factors

Core NMD factors SMG1‐7 were initially discovered using genetic screens in *C. elegans* and in *S. cerevisiae* and were later found by homology searches in other species, including *Arabidopsis*, *Drosophila*, and mammals. Since then, a variety of experimental approaches have been employed to identify novel regulators of this pathway, most of which are still being investigated and therefore do not feature in models of NMD (Table 1) (Hug *et al*, 2016).

**Table 1:** Factors involved in nonsense‐mediated mRNA decay.

Protein	Role in nonsense‐mediated mRNA decay	References
SMG1	A phosphoinositide 3‐kinase (PI3K)‐like kinase that phosphorylates UPF1	Yamashita *et al* (2001), Kashima *et al* (2006)
SMG8/9	Two subunits of the SMG‐1 complex which negatively regulate SMG‐1 kinase activity	Yamashita *et al* (2009)
UPF1	An ATP‐dependent RNA helicase of the SF1 superfamily, which undergoes cycles of phosphorylation and dephosphorylation	Sun *et al* (1998), Bhattacharya *et al* (2000), Kim and Maquat (2019)
UPF2	Recruited following recognition of a PTC leading to conformational changes in UPF1 and formation of the DECID complex	Serin *et al* (2001), Chamieh *et al* (2008)
UPF3A/B	UPF3 paralogs that bridge the EJC and the surveillance complex	Serin *et al* (2001), Neu‐Yilik *et al* (2017)
SMG5/7	A heterodimer that binds to phosphorylated UPF1 and recruits the exonucleolytic RNA degrading machinery	Unterholzner and Izaurralde (2004), Loh *et al* (2013)
SMG6	An endonuclease that cleaves NMD targets in the vicinity of the PTC	Huntzinger *et al* (2008), Eberle *et al* (2009)
DHX34	A DExH/D box helicase that activates NMD by promoting a transition from the SURF to DECID complex	Hug and Cáceres (2014)
NBAS	NMD factor localized at the membrane of the ER that recruits UPF1 to activate a local NMD response	Longman *et al* (2007, 2020)
MOV10	A 5′ to 3′ RNA helicase that contributes to UPF1‐mediated mRNA target degradation	Gregersen *et al* (2014)
RUVBL1/2	AAA+ adenosine triphosphatases involved in the early stages of NMD that interact with SMG‐1 and promote the formation of the SURF complex	Izumi *et al* (2010)
ICE1	An EJC‐associated protein that promotes UPF3B recruitment to the EJC and provides a link between splicing and NMD	Baird *et al* (2018)
GIGYF2/EIF4E2	Mediate translational repression following recognition of a PTC	Zinshteyn *et al* (2021)
AKT	AKT signaling leads to formation of alternative EJCs where AKT replaces UPF2. AKT‐mediated phosphorylation of UPF1 activates NMD.	Palma *et al* (2021), Cho *et al* (2022)
CWC22	Essential splicing factor which interacts with eIFA3 to activate NMD	Alexandrov *et al* (2012), Barbosa *et al* (2012), Steckelberg *et al* (2012)
ABCE1	Responsible for ribosome recycling necessary to initiate translation termination and initiation of NMD via SMG6‐mediated endonucleolytic pathway	Annibaldis *et al* (2020), Zhu *et al* (2020)

We developed RNAi screens in *C. elegans* that resulted in the identification of novel NMD factors, including *smgl‐2/DHX34* and *smgl‐1/NBAS* that are conserved throughout evolution and function in NMD in nematodes, zebrafish, and human cells (Longman *et al*, 2007, 2013; Anastasaki *et al*, 2011). DHX34, a DExH/D box RNA helicase, forms a complex with SMG1 and UPF1 and activates NMD by promoting the transition from the surveillance to the decay‐inducing complex (Hug & Cáceres, 2014; Melero *et al*, 2016). Heterozygous mutations in *DHX34* were identified in four families affected with inherited acute myeloid leukemia (AML) and myelodysplastic syndrome (MDS). These mutations map to different domains of DHX34, and all germline variants identified in these families abrogated NMD activity (Rio‐Machin *et al*, 2020). DHX34 is also associated with the human spliceosomal catalytic C complex and regulates a large number of alternative splicing (AS) events in mammalian cells in culture, establishing a dual role for DHX34 in both NMD and pre‐mRNA splicing (Hug *et al*, 2022).

Neuroblastoma amplified sequence (*NBAS*) encodes a protein localized to the endoplasmic reticulum (ER) that is a component of the Syntaxin 18 complex and fulfils a role in Golgi‐to ER retrograde transport, which is independent of its function in NMD (Aoki *et al*, 2009; Longman *et al*, 2020; discussed below in the “Localized NMD and the stress response” section).

Interactome studies, RNAi screens, and newer CRISPR screens in mammalian cells in culture have led to the identification of novel NMD regulators, which are still being characterized (Table 1). The RNA helicase MOV10, a member of the UPF1‐like group helicase superfamily 1 (SF1), interacts with UPF1 and promotes degradation of UPF1‐regulated mRNA transcripts (Gregersen *et al*, 2014). An interactome of the SMG1 protein kinase identified the AAA ATPases, RuVB‐like 1 (RUVBL1) and RuVB‐like 2 (RUVBL2). These proteins are involved in a variety of cellular functions, including transcription and DNA repair, and were previously shown to have a role in the early stages of NMD (Izumi *et al*, 2010). We recently showed that RUVBL1/2 also interact with DHX34, coupling their ATPase activity to the assembly of factors required to initiate the NMD response (López‐Perrote *et al*, 2020).

A genome‐wide RNAi screen in human cells identified ICE1, an EJC‐associated factor that promotes the interaction of UPF3B with the EJC and activates NMD (Baird *et al*, 2018). CRISPR screens identified several candidate NMD genes (Alexandrov *et al*, 2017) and highlighted a role for the ribosome recycling factor ABCE1 in NMD (Annibaldis *et al*, 2020; Zhu *et al*, 2020). A CRISPR screen in K562 cells identified the translational repressors GIGYF2 and EIF4E2, suggesting a model wherein recognition of a stop codon as premature leads to its translational repression mediated by GIGYF2 and EIF4E2, a process shared with the NGD pathway (Zinshteyn *et al*, 2021). Finally, a haploid‐cell genetic screen for NMD effectors identified several components of the AKT signaling pathway. It was shown that AKT‐mediated phosphorylation of the UPF1 CH domain at T151D overcomes auto‐inhibition of UPF1 helicase activity, which is critical for NMD and decreases the dependence of helicase activity on ATP. AKT also promotes formation of EJCs that contain AKT at the expense of UPF2, potentially facilitating a UPF2 independent branch of NMD (Cho *et al*, 2022). Interestingly, AKT1 had been independently shown to phosphorylate UPF1 and activate NMD (Palma *et al*, 2021; Table 1).

NMD factors have also been shown to display additional cellular functions. This is particularly prominent in the case of UPF1 that not only functions in genome stability (Azzalin & Lingner, 2006), but also contributes to several RNA decay pathways (Kim & Maquat, 2019). These include the Staufen‐mediated decay pathway (SMD), which involves UPF1 recruitment to stem‐loops in the 3′UTRs of mRNAs bound by the RNA‐binding proteins STAUFEN1 and its paralog STAUFEN 2 (Kim *et al*, 2005; Park *et al*, 2013), and in the degradation of histone mRNAs, where UPF1 is recruited to target mRNAs via the stem‐loop binding protein (SLBP) (Kaygun & Marzluff, 2005). Interestingly, UPF1 has also been proposed to act as an E3‐ubiquitin ligase and promote degradation of the truncated polypeptide produced by translation of a PTC‐containing transcript (Takahashi *et al*, 2008; Feng *et al*, 2017), establishing a link between RNA degradation and protein decay (Kim & Maquat, 2019; Inglis *et al*, 2023). In *S. cerevisiae*, UPF1 facilitates proteasome degradation of truncated polypeptides in a ubiquitin dependent manner. The truncated protein product gets released from the ribosome following NMD‐mediated mRNA degradation, but remains associated with UPF1 which directs it to the proteasome for removal, thus leading to a protein and mRNA turnover coupled process (Kuroha *et al*, 2013).

### NMD targets

Despite decades of research, it is still not entirely clear what constitutes a *bona fide* NMD target. The introduction of a mutation, such as a single nucleotide variant, which gives rise to a PTC, represents the most obvious target for NMD. However, transcriptome profiling to identify NMD targets in cells of different species revealed that the majority of NMD‐sensitive transcripts do not contain PTCs but are rather mRNAs coding for full‐length proteins. This led to the hypothesis that a combination of NMD‐inducing and NMD‐antagonizing features will contribute to determine NMD susceptibility for any given mRNA. Some of the common features that render mRNAs susceptible to NMD include the presence of a PTC located at least 50‐55 nucleotides upstream of an EJC (Colombo *et al*, 2017), mRNAs with upstream ORFs (uORFs; Calvo *et al*, 2009), and the presence of a long 3′UTR (Hogg & Goff, 2010). However, recent use of cDNA Nanopore sequencing combined with short RNA‐seq allowed the detection of full‐length NMD substrates that are highly unstable and only display an increase in RNA levels when NMD is inhibited. This analysis identified NMD target mRNAs derived mainly from alternative exon usage, yet it did not identify long 3′UTRs as a common feature for NMD regulated mRNAs (Karousis *et al*, 2021). RNA‐seq allows for the analysis of steady‐state changes, which are influenced by stability, degradation, and transcription rates. Recently, SH‐linked alkylation for the metabolic sequencing of RNA (SLAM‐seq) using 4‐thiouracil pulse‐chase labeling (Herzog *et al*, 2017) was used to accurately measure changes in RNA half‐lives and to identify new targets of the NMD pathway in *S. cerevisiae* (Alalam *et al*, 2022). SLAM seq analysis of *Smg*5‐7 genetic knockouts in mouse ESCs revealed that NMD controls expression levels of the translation initiation factor *Eif4a2* and its alternative splicing isoform that harbors a PTC‐encoding isoform (*Eif4a2* ^*PTC*^). Upon NMD inhibition, aberrant expression of the eIF4A2^PTC^ elicits increased mTORC1 activity and translation rates and causes differentiation delays, highlighting a role of RNA stability regulation in development (Huth *et al*, 2022).

### NMD regulation

The NMD pathway is dynamic and subject to regulation and impacts several physiological processes, such as the stress and immune responses. A correct magnitude of NMD activity is particularly important for proper brain function and indeed, NMD activity is extensively regulated during neural development (Jaffrey & Wilkinson, 2018). It was shown that NMD activity in human neuroblastoma cells is attenuated by fragile X protein FMRP, which is recruited to NMD targets by UPF1. FMRP acts as an NMD repressor in neural cells and in its absence, NMD is hyperactivated, leading to widespread transcriptome changes which contributes to intellectual disability and autism (Kurosaki *et al*, 2021). Recently, a conditional *Smg6* mutant mouse model revealed that the NMD pathway has a role in controlling circadian clock regulation (Katsioudi *et al*, 2023). The NMD response varies within and across cell lines (Sato & Singer, 2021), in different cell tissues (Zetoune *et al*, 2008), and even among individuals (Nguyen *et al*, 2014; Rivas *et al*, 2015). NMD is tightly regulated by a negative feedback network that leads to a large proportion of core NMD factors being regulated by NMD itself in several organisms, including mammalian cells, nematodes, zebrafish, and plants (Huang *et al*, 2011; Yepiskoposyan *et al*, 2011; Longman *et al*, 2013). NMD can occur with equal probability during each round of translation of an mRNA molecule; however, this probability is variable and linked to sequence features, including the exon sequence downstream of the PTC, the PTC‐to‐intron distance, and the number of introns both upstream and downstream of the PTC. Furthermore, a subpopulation of mRNAs can escape NMD, further contributing to variation in NMD efficiency (Hoek *et al*, 2019). Use of a single‐cell approach comprising a bi‐directional NMD reporter expressing two β‐globin genes with or without a PTC in the same cell, allowed the characterization NMD efficiency in individual cells. This revealed a broad range of NMD efficiencies in the population (where some cells degraded essentially all mRNAs and others escaped NMD almost completely) and was correlated to the differential level of SMG1 expression and P‐UPF1. Mechanistically, this escape occurred either by translational read‐through at the PTC or by inefficient mRNA degradation following translation termination at the PTC (Sato & Singer, 2021).

### Localized NMD and the stress response

**Figure 5:**
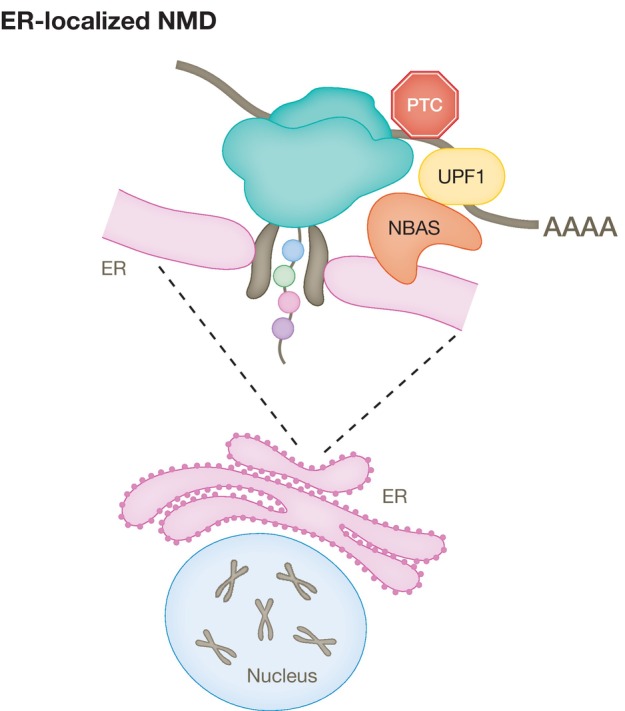
Localized NMD response at the ER NBAS localizes to the outside membrane of the endoplasmic reticulum (ER), in the vicinity of the translocon, where it recruits the core NMD factor UPF1 to activate a local NMD response at the ER (Longman *et al*, 2020).

The decay of NMD reporters in mammalian cells occurs in the cytoplasm (Trcek *et al*, 2013) and is closely linked to mRNA translation (Kervestin & Jacobson, 2012). We previously identified two novel NMD factors, *NBAS* and *SEC13*, which localize to the ER, raising the possibility that they could be involved in a localized NMD pathway (Longman *et al*, 2007; Casadio *et al*, 2015). There are precedents for a localized NMD response in neurons that regulate the expression of dendritic and axonal mRNAs upon the activation of their localized mRNA translation (Colak *et al*, 2013). It has been shown that mRNAs coding for secreted or transmembrane proteins are translated only when they encounter the ER (Wu *et al*, 2016). However, it remained largely unknown how NMD regulates the stability of RNAs translated at the ER, which due to their intrinsic localized translation, will not have sufficient exposure to cytoplasmic NMD surveillance. NBAS is a member of the Syntaxin 18 complex as part of the COPI vesicle and is involved in Golgi‐to‐ER retrograde transport (Aoki *et al*, 2009). We showed that NBAS fulfils a second, independent function and recruits the core NMD factor UPF1 to the membrane of the ER and activates a local NMD response that regulates RNAs associated with cellular stress and membrane trafficking (Fig 5; Longman *et al*, 2020). Loss‐of‐function mutations in NBAS have been found in diseases affecting bone, connective tissue, and acute liver failure (Hug *et al*, 2016; Staufner *et al*, 2020). We identified compound heterozygous variants in *NBAS* as a cause of atypical osteogenesis imperfecta (Balasubramanian *et al*, 2017). It remains to be seen whether the phenotype of *NBAS* mutations is due to faulty NMD response and/or defective Golgi‐to‐ER retrograde transport (Haack *et al*, 2015).

The Unfolded Protein Response (UPR) senses and responds to excessive amounts of misfolded proteins in the ER, that cause ER stress (Walter & Ron, 2011). Inappropriate UPR activation contributes to many human pathologies, most notably related to neurodegeneration (Hetz & Mollereau, 2014), therefore the fidelity of UPR activation must be tightly regulated. Accordingly, a role for NMD in regulating the UPR has been proposed (Karam *et al*, 2015; Goetz & Wilkinson, 2017). In particular, mRNAs encoding UPR components, including the UPR sensor IRE1α, as well as ATF‐4 and CHOP that are activated by PERK branch signaling, are regulated by NMD and thus control the threshold of cellular stress that is necessary to activate the UPR (Gardner, 2008; Karam *et al*, 2015; Sieber *et al*, 2016). A feed‐back loop mechanism operates, wherein NMD ensures a correct activation threshold for the UPR response and the activity of NMD is in turn down‐regulated by the UPR (Karam *et al*, 2015). It is likely that limiting the chronic activation of the UPR has a protective effect in neurodegenerative diseases. Along those lines, a protective role for UPF1 was shown in rodent primary neuronal models of amyotrophic lateral sclerosis (ALS) and frontotemporal dementia (FTD; Barmada *et al*, 2015) and in a rat ALS paralysis model (Jackson *et al*, 2015).

It is also possible that modulation of ER‐NMD activity could be crucial to regulate ER stress (Longman *et al*, 2020). In such a scenario, NMD‐mediated degradation of RNA targets would decrease ER load by suppressing aggregation of truncated and/or misfolded proteins in the ER, limiting UPR activation. The newly identified UPF1_LL_ that, upon ISR activation and impaired translation, regulates a new subset of NMD targets, can be a key to the appropriate cellular stress response (Fritz *et al*, 2022).

### NMD in human disease

In vertebrates, NMD activity is essential for proper development, and loss of NMD leads to embryonic lethality (Chousal *et al*, 2022). There are several reports of mutations in core NMD factors that lead to human disease. Two consanguineous families with homozygous loss‐of‐function mutations in SMG9, a component of the SMG1c complex, display a multiple congenital anomaly syndrome, that includes heart and eye defects, and brain malformation (Shaheen *et al*, 2016). Four consanguineous families with four deleterious homozygous variants in SMG8 display a phenotype that resembles that found in patients with SMG9 mutations, including global developmental delay, microcephaly, facial dysmorphism, and variable congenital heart and eye malformations. These patients display increased phosphorylation of UPF1, which most likely reflect the loss of SMG8‐mediated inhibition of SMG1 kinase activity (Alzahrani *et al*, 2020). NMD has also an important role in neuronal development as demonstrated by the fact that mutations, as well as copy number variations in UPF2 and UPF3B, lead to intellectual disability and/or neurodevelopmental disorders in humans, including schizophrenia and autism spectrum disorder (Jolly *et al*, 2013; Nguyen *et al*, 2013; Jaffrey & Wilkinson, 2018). Altered levels of NMD activity were also implicated in the pathogenesis of C9orf72‐linked ALS/FTD (Xu *et al*, 2019; Ortega *et al*, 2020; Sun *et al*, 2020) and other neurodegenerative diseases (Kurosaki *et al*, 2021). For example, loss of the fragile X protein FMRP leads to intellectual disability and autism, and FMRP deficiency results in a hyperactivated NMD response in human cells (Kurosaki *et al*, 2021). UPF3B mutations cause intellectual disability with impairment of neural stem cell differentiation and reduction in neuronal branching, through a loss of UPF2 interaction leading to NMD abrogation (Bufton *et al*, 2022). Altogether, these data suggest that an imbalance in NMD activity could lead to neurodegeneration. Interestingly, NGD and NSD components *Pelo* and *HBs1l* are critical for cerebellum neurogenesis in mice but expendable for survival of these neurons after development (Terrey *et al*, 2021). Similar effects were observed upon deletion of the core NMD factor, Upf2. This suggests that several RNA quality control pathways may interact or have compensatory roles to drive early development.

The role of NMD in cancer is complex, since it can display both tumor suppressing and tumor enhancing roles (Wang *et al*, 2011; Tan *et al*, 2022). Cancer cells can exploit the NMD response through introduction of selective tumor suppressor mutations that initiate mRNA decay or via the introduction of NMD‐insensitive mutations in oncogenes to prevent their targeting (Lindeboom *et al*, 2016). For example, tumor cells are known to hijack the NMD system to suppress the expression of potent tumor suppressors such as WT1, BRCA1/2 and p53, inducing uncontrolled cell growth (Mort *et al*, 2008; Nogueira *et al*, 2021). A therapeutic potential for the treatment of cancer was suggested in a number of studies. Abrogating NMD in cancer may lead to the expression of tumor‐specific proteins that can increase natural immune responses directed against the tumor (Pastor *et al*, 2010). Furthermore, attenuation of NMD facilitates the response to cancer therapeutics, as shown in human breast cancer cells, subject to NMD inhibition. Combining this with the front‐line chemotherapeutic doxorubicin promotes a faster and more robust cancer‐cell killing by apoptosis (Popp & Maquat, 2015).

## Conclusions

Translation‐coupled RNA quality control pathways play a central role in ensuring accurate gene expression. There is a great variety of such mechanisms that link defective mRNA translation with mRNA decay and degradation of truncated proteins. We have highlighted here three main pathways, NGD, NSD, and NMD. The first two share common features and trans‐acting factors and mainly differ in features present in the target mRNAs that trigger these pathways. NMD is the most studied mRNA surveillance system, both in terms of mechanism and trans‐acting factors, mostly due to its high impact as a potent buffering system for human disease. Given that the NMD pathway affects the phenotype of approximately one third of all genetic diseases (Holbrook *et al*, 2004; Mort *et al*, 2008), a better understanding of its regulation and factors that influence its function *in vivo* could be of importance for designing strategies to modulate the NMD response for therapeutic use. Challenges for the future involve the identification of all factors required for NMD regulation in a physiological setting, as well as a better understanding of what constitutes an NMD target.

## Author contributions

**Javier F Caceres:** Conceptualization; writing – original draft; writing – review and editing. **Laura Monaghan:** Conceptualization; writing – original draft; writing – review and editing. **Dasa Longman:** Conceptualization; writing – original draft; writing – review and editing.

## Disclosure and competing interests statement

Javier F Caceres is a member of the Advisory Editorial Board of The EMBO Journal. This has no bearing on the editorial consideration of this article for publication.

## References

[embj2023114378-bib-0001] Alalam H , Zepeda‐Martínez JA , Sunnerhagen P (2022) Global SLAM‐seq for accurate mRNA decay determination and identification of NMD targets. RNA 28: 905–915 35296539 10.1261/rna.079077.121 PMC9074897

[embj2023114378-bib-0002] Alexandrov A , Colognori D , Shu M‐D , Steitz JA (2012) Human spliceosomal protein CWC22 plays a role in coupling splicing to exon junction complex deposition and nonsense‐mediated decay. Proc Natl Acad Sci USA 109: 21313–21318 23236153 10.1073/pnas.1219725110 PMC3535618

[embj2023114378-bib-0003] Alexandrov A , Shu M‐D , Steitz JA (2017) Fluorescence amplification method for forward genetic discovery of factors in human mRNA degradation. Mol Cell 65: 191–201 28017590 10.1016/j.molcel.2016.11.032 PMC5301997

[embj2023114378-bib-0004] Alzahrani F , Kuwahara H , Long Y , Al‐Owain M , Tohary M , AlSayed M , Mahnashi M , Fathi L , Alnemer M , Al‐Hamed MH *et al* (2020) Recessive, deleterious variants in SMG8 expand the role of nonsense‐mediated decay in developmental disorders in humans. Am J Hum Genet 107: 1178–1185 33242396 10.1016/j.ajhg.2020.11.007 PMC7820624

[embj2023114378-bib-0005] Anastasaki C , Longman D , Capper A , Patton EE , Cáceres JF (2011) Dhx34 and Nbas function in the NMD pathway and are required for embryonic development in zebrafish. Nucleic Acids Res 39: 3686–3694 21227923 10.1093/nar/gkq1319 PMC3089463

[embj2023114378-bib-0006] Annibaldis G , Domanski M , Dreos R , Contu L , Carl S , Klaÿ N , Mühlemann O (2020) Readthrough of stop codons under limiting ABCE1 concentration involves frameshifting and inhibits nonsense‐mediated mRNA decay. Nucleic Acids Res 48: 10259–10279 32941650 10.1093/nar/gkaa758 PMC7544199

[embj2023114378-bib-0007] Aoki T , Ichimura S , Itoh A , Kuramoto M , Shinkawa T , Isobe T , Tagaya M (2009) Identification of the neuroblastoma‐amplified gene product as a component of the syntaxin 18 complex implicated in Golgi‐to‐endoplasmic reticulum retrograde transport. Mol Biol Cell 20: 2639–2649 19369418 10.1091/mbc.E08-11-1104 PMC2688544

[embj2023114378-bib-0008] Azzalin CM , Lingner J (2006) The human RNA surveillance factor UPF1 is required for S phase progression and genome stability. Curr Biol 16: 433–439 16488880 10.1016/j.cub.2006.01.018

[embj2023114378-bib-0009] Baird TD , Cheng KC‐C , Chen Y‐C , Buehler E , Martin SE , Inglese J , Hogg JR (2018) ICE1 promotes the link between splicing and nonsense‐mediated mRNA decay. eLife 7: e33178 29528287 10.7554/eLife.33178 PMC5896957

[embj2023114378-bib-0010] Balasubramanian M , Hurst J , Brown S , Bishop NJ , Arundel P , DeVile C , Pollitt RC , Crooks L , Longman D , Caceres JF *et al* (2017) Compound heterozygous variants in NBAS as a cause of atypical osteogenesis imperfecta. Bone 94: 65–74 27789416 10.1016/j.bone.2016.10.023 PMC6067660

[embj2023114378-bib-0011] Barbosa I , Haque N , Fiorini F , Barrandon C , Tomasetto C , Blanchette M , Le Hir H (2012) Human CWC22 escorts the helicase eIF4AIII to spliceosomes and promotes exon junction complex assembly. Nat Struct Mol Biol 19: 983–990 22961380 10.1038/nsmb.2380

[embj2023114378-bib-0012] Barmada SJ , Ju S , Arjun A , Batarse A , Archbold HC , Peisach D , Li X , Zhang Y , Tank EMH , Qiu H *et al* (2015) Amelioration of toxicity in neuronal models of amyotrophic lateral sclerosis by hUPF1. Proc Natl Acad Sci USA 112: 7821–7826 26056265 10.1073/pnas.1509744112 PMC4485101

[embj2023114378-bib-0013] Bhattacharya A , Czaplinski K , Trifillis P , He F , Jacobson A , Peltz SW (2000) Characterization of the biochemical properties of the human Upf1 gene product that is involved in nonsense‐mediated mRNA decay. RNA 6: 1226–1235 10999600 10.1017/s1355838200000546 PMC1369996

[embj2023114378-bib-0014] Bhuvanagiri M , Schlitter AM , Hentze MW , Kulozik AE (2010) NMD: RNA biology meets human genetic medicine. Biochem J 430: 365–377 20795950 10.1042/BJ20100699

[embj2023114378-bib-0015] Boehm V , Kueckelmann S , Gerbracht JV , Kallabis S , Britto‐Borges T , Altmüller J , Krüger M , Dieterich C , Gehring NH (2021) SMG5‐SMG7 authorize nonsense‐mediated mRNA decay by enabling SMG6 endonucleolytic activity. Nat Commun 12: 3965 34172724 10.1038/s41467-021-24046-3 PMC8233366

[embj2023114378-bib-0016] Brandman O , Hegde RS (2016) Ribosome‐associated protein quality control. Nat Struct Mol Biol 23: 7–15 26733220 10.1038/nsmb.3147 PMC4853245

[embj2023114378-bib-0017] Bufton JC , Powers KT , Szeto JA , Toelzer C , Berger I , Schaffitzel C (2022) Structures of nonsense‐mediated mRNA decay factors UPF3B and UPF3A in complex with UPF2 reveal molecular basis for competitive binding and for neurodevelopmental disorder‐causing mutation. Nucleic Acids Res 50: 5934–5947 35640974 10.1093/nar/gkac421 PMC9177958

[embj2023114378-bib-0018] Bühler M , Steiner S , Mohn F , Paillusson A , Mühlemann O (2006) EJC‐independent degradation of nonsense immunoglobulin‐mu mRNA depends on 3′ UTR length. Nat Struct Mol Biol 13: 462–464 16622410 10.1038/nsmb1081

[embj2023114378-bib-0019] Cacciottolo M , Numitone G , Aurino S , Caserta IR , Fanin M , Politano L , Minetti C , Ricci E , Piluso G , Angelini C *et al* (2011) Muscular dystrophy with marked Dysferlin deficiency is consistently caused by primary dysferlin gene mutations. Eur J Hum Genet 19: 974–980 21522182 10.1038/ejhg.2011.70 PMC3179367

[embj2023114378-bib-0020] Calvo SE , Pagliarini DJ , Mootha VK (2009) Upstream open reading frames cause widespread reduction of protein expression and are polymorphic among humans. Proc Natl Acad Sci USA 106: 7507–7512 19372376 10.1073/pnas.0810916106 PMC2669787

[embj2023114378-bib-0021] Casadio A , Longman D , Hug N , Delavaine L , Vallejos Baier R , Alonso CR , Cáceres JF (2015) Identification and characterization of novel factors that act in the nonsense‐mediated mRNA decay pathway in nematodes, flies and mammals. EMBO Rep 16: 71–78 25452588 10.15252/embr.201439183 PMC4304730

[embj2023114378-bib-0022] Chakrabarti S , Jayachandran U , Bonneau F , Fiorini F , Basquin C , Domcke S , Le Hir H , Conti E (2011) Molecular mechanisms for the RNA‐dependent ATPase activity of Upf1 and its regulation by Upf2. Mol Cell 41: 693–703 21419344 10.1016/j.molcel.2011.02.010

[embj2023114378-bib-0023] Chamieh H , Ballut L , Bonneau F , Le Hir H (2008) NMD factors UPF2 and UPF3 bridge UPF1 to the exon junction complex and stimulate its RNA helicase activity. Nat Struct Mol Biol 15: 85–93 18066079 10.1038/nsmb1330

[embj2023114378-bib-0024] Chan W‐K , Huang L , Gudikote JP , Chang Y‐F , Imam JS , MacLean JA , Wilkinson MF (2007) An alternative branch of the nonsense‐mediated decay pathway. EMBO J 26: 1820–1830 17363904 10.1038/sj.emboj.7601628 PMC1847659

[embj2023114378-bib-0025] Chandrasekaran V , Juszkiewicz S , Choi J , Puglisi JD , Brown A , Shao S , Ramakrishnan V , Hegde RS (2019) Mechanism of ribosome stalling during translation of a poly(A) tail. Nat Struct Mol Biol 26: 1132–1140 31768042 10.1038/s41594-019-0331-x PMC6900289

[embj2023114378-bib-0026] Chapman JH , Craig JM , Wang CD , Gundlach JH , Neuman KC , Hogg JR (2022) UPF1 mutants with intact ATPase but deficient helicase activities promote efficient nonsense‐mediated mRNA decay. Nucleic Acids Res 50: 11876–11894 36370101 10.1093/nar/gkac1026 PMC9723629

[embj2023114378-bib-0027] Chen C , Shen Y , Li L , Ren Y , Wang Z‐Q , Li T (2023) UPF3A is dispensable for nonsense‐mediated mRNA decay in mouse pluripotent and somatic cells. Life Sci Alliance 6: e202201589 36997282 10.26508/lsa.202201589 PMC10070813

[embj2023114378-bib-0028] Cho H , Abshire ET , Popp MW , Pröschel C , Schwartz JL , Yeo GW , Maquat LE (2022) AKT constitutes a signal‐promoted alternative exon‐junction complex that regulates nonsense‐mediated mRNA decay. Mol Cell 82: 2779–2796.e10 35675814 10.1016/j.molcel.2022.05.013 PMC9357146

[embj2023114378-bib-0029] Chousal JN , Sohni A , Vitting‐Seerup K , Cho K , Kim M , Tan K , Porse B , Wilkinson MF , Cook‐Andersen H (2022) Progression of the pluripotent epiblast depends upon the NMD factor UPF2. Development 149: dev200764 36255229 10.1242/dev.200764 PMC9687065

[embj2023114378-bib-0030] Colak D , Ji S‐J , Porse BT , Jaffrey SR (2013) Regulation of axon guidance by compartmentalized nonsense‐mediated mRNA decay. Cell 153: 1252–1265 23746841 10.1016/j.cell.2013.04.056 PMC3685487

[embj2023114378-bib-0031] Colombo M , Karousis ED , Bourquin J , Bruggmann R , Mühlemann O (2017) Transcriptome‐wide identification of NMD‐targeted human mRNAs reveals extensive redundancy between SMG6‐ and SMG7‐mediated degradation pathways. RNA 23: 189–201 27864472 10.1261/rna.059055.116 PMC5238794

[embj2023114378-bib-0032] D’Orazio KN , Green R (2021) Ribosome states signal RNA quality control. Mol Cell 81: 1372–1383 33713598 10.1016/j.molcel.2021.02.022 PMC8041214

[embj2023114378-bib-0033] D’Orazio KN , Wu CC‐C , Sinha N , Loll‐Krippleber R , Brown GW , Green R (2019) The endonuclease Cue2 cleaves mRNAs at stalled ribosomes during No Go Decay. Elife 8: e49117 31219035 10.7554/eLife.49117 PMC6598757

[embj2023114378-bib-0034] Dave P , Roth G , Griesbach E , Mateju D , Hochstoeger T , Chao JA (2023) Single‐molecule imaging reveals translation‐dependent destabilization of mRNAs. Mol Cell 83: 589–606.e6 36731471 10.1016/j.molcel.2023.01.013 PMC9957601

[embj2023114378-bib-0035] Doma MK , Parker R (2006) Endonucleolytic cleavage of eukaryotic mRNAs with stalls in translation elongation. Nature 440: 561–564 16554824 10.1038/nature04530 PMC1839849

[embj2023114378-bib-0036] Eberle AB , Lykke‐Andersen S , Mühlemann O , Jensen TH (2009) SMG6 promotes endonucleolytic cleavage of nonsense mRNA in human cells. Nat Struct Mol Biol 16: 49–55 19060897 10.1038/nsmb.1530

[embj2023114378-bib-0037] Feng Q , Jagannathan S , Bradley RK (2017) The RNA surveillance factor UPF1 represses myogenesis via its E3 ubiquitin ligase activity. Mol Cell 67: 239–251.e6 28669802 10.1016/j.molcel.2017.05.034 PMC5536975

[embj2023114378-bib-0038] Fernández IS , Yamashita A , Arias‐Palomo E , Bamba Y , Bartolomé RA , Canales MA , Teixidó J , Ohno S , Llorca O (2011) Characterization of SMG‐9, an essential component of the nonsense‐mediated mRNA decay SMG1C complex. Nucleic Acids Res 39: 347–358 20817927 10.1093/nar/gkq749 PMC3017601

[embj2023114378-bib-0039] Fiorini F , Bagchi D , Le Hir H , Croquette V (2015) Human Upf1 is a highly processive RNA helicase and translocase with RNP remodelling activities. Nat Commun 6: 7581 26138914 10.1038/ncomms8581 PMC4506499

[embj2023114378-bib-0040] Franks TM , Singh G , Lykke‐Andersen J (2010) Upf1 ATPase‐dependent mRNP disassembly is required for completion of nonsense‐mediated mRNA decay. Cell 143: 938–950 21145460 10.1016/j.cell.2010.11.043 PMC3357093

[embj2023114378-bib-0041] Frischmeyer PA , van Hoof A , O’Donnell K , Guerrerio AL , Parker R , Dietz HC (2002) An mRNA surveillance mechanism that eliminates transcripts lacking termination codons. Science 295: 2258–2261 11910109 10.1126/science.1067338

[embj2023114378-bib-0042] Fritz SE , Ranganathan S , Wang CD , Hogg JR (2022) An alternative UPF1 isoform drives conditional remodeling of nonsense‐mediated mRNA decay. EMBO J 41: e108898 35403729 10.15252/embj.2021108898 PMC9108617

[embj2023114378-bib-0043] Ganesan R , Mangkalaphiban K , Baker R , He F , Jacobson A (2022) Ribosome‐bound Upf1 forms distinct 80S complexes and conducts mRNA surveillance. RNA 28: 1621–1642 36192133 10.1261/rna.079416.122 PMC9670811

[embj2023114378-bib-0044] Gardner LB (2008) Hypoxic inhibition of nonsense‐mediated RNA decay regulates gene expression and the integrated stress response. Mol Cell Biol 28: 3729–3741 18362164 10.1128/MCB.02284-07 PMC2423288

[embj2023114378-bib-0045] Gehring NH , Kunz JB , Neu‐Yilik G , Breit S , Viegas MH , Hentze MW , Kulozik AE (2005) Exon‐junction complex components specify distinct routes of nonsense‐mediated mRNA decay with differential cofactor requirements. Mol Cell 20: 65–75 16209946 10.1016/j.molcel.2005.08.012

[embj2023114378-bib-0046] Glover ML , Burroughs AM , Monem PC , Egelhofer TA , Pule MN , Aravind L , Arribere JA (2020) NONU‐1 encodes a conserved endonuclease required for mRNA translation surveillance. Cell Rep 30: 4321–4331.e4 32234470 10.1016/j.celrep.2020.03.023 PMC7184879

[embj2023114378-bib-0047] Goetz AE , Wilkinson M (2017) Stress and the nonsense‐mediated RNA decay pathway. Cell Mol Life Sci 74: 3509–3531 28503708 10.1007/s00018-017-2537-6 PMC5683946

[embj2023114378-bib-0048] Gowravaram M , Bonneau F , Kanaan J , Maciej VD , Fiorini F , Raj S , Croquette V , Le Hir H , Chakrabarti S (2018) A conserved structural element in the RNA helicase UPF1 regulates its catalytic activity in an isoform‐specific manner. Nucleic Acids Res 46: 2648–2659 29378013 10.1093/nar/gky040 PMC5861435

[embj2023114378-bib-0049] Gregersen LH , Schueler M , Munschauer M , Mastrobuoni G , Chen W , Kempa S , Dieterich C , Landthaler M (2014) MOV10 Is a 5′ to 3′ RNA helicase contributing to UPF1 mRNA target degradation by translocation along 3′ UTRs. Mol Cell 54: 573–585 24726324 10.1016/j.molcel.2014.03.017

[embj2023114378-bib-0050] Haack TB , Staufner C , Köpke MG , Straub BK , Kölker S , Thiel C , Freisinger P , Baric I , McKiernan PJ , Dikow N *et al* (2015) Biallelic mutations in NBAS cause recurrent acute liver failure with onset in infancy. Am J Hum Genet 97: 163–169 26073778 10.1016/j.ajhg.2015.05.009 PMC4572578

[embj2023114378-bib-0051] Hall GW , Thein S (1994) Nonsense codon mutations in the terminal exon of the beta‐globin gene are not associated with a reduction in beta‐mRNA accumulation: a mechanism for the phenotype of dominant beta‐thalassemia. Blood 83: 2031–2037 8161774

[embj2023114378-bib-0052] Hanson G , Coller J (2018) Codon optimality, bias and usage in translation and mRNA decay. Nat Rev Mol Cell Biol 19: 20–30 29018283 10.1038/nrm.2017.91 PMC6594389

[embj2023114378-bib-0053] Harigaya Y , Parker R (2010) No‐go decay: a quality control mechanism for RNA in translation. Wiley Interdiscip Rev RNA 1: 132–141 21956910 10.1002/wrna.17

[embj2023114378-bib-0054] He F , Jacobson A (2015) Nonsense‐mediated mRNA decay: degradation of defective transcripts is only part of the story. Annu Rev Genet 49: 339–366 26436458 10.1146/annurev-genet-112414-054639 PMC4837945

[embj2023114378-bib-0055] Herzog VA , Reichholf B , Neumann T , Rescheneder P , Bhat P , Burkard TR , Wlotzka W , Von Haeseler A , Zuber J , Ameres SL (2017) Thiol‐linked alkylation of RNA to assess expression dynamics. Nat Methods 14: 1198–1204 28945705 10.1038/nmeth.4435 PMC5712218

[embj2023114378-bib-0056] Hetz C , Mollereau B (2014) Disturbance of endoplasmic reticulum proteostasis in neurodegenerative diseases. Nat Rev Neurosci 15: 233–249 24619348 10.1038/nrn3689

[embj2023114378-bib-0057] Hickey KL , Dickson K , Cogan JZ , Replogle JM , Schoof M , D’Orazio KN , Sinha NK , Hussmann JA , Jost M , Frost A *et al* (2020) GIGYF2 and 4EHP inhibit translation initiation of defective messenger RNAs to assist ribosome‐associated quality control. Mol Cell 79: 950–962.e6 32726578 10.1016/j.molcel.2020.07.007 PMC7891188

[embj2023114378-bib-0058] Hoek TA , Khuperkar D , Lindeboom RGH , Sonneveld S , Verhagen BMP , Boersma S , Vermeulen M , Tanenbaum ME (2019) Single‐molecule imaging uncovers rules governing nonsense‐mediated mRNA decay. Mol Cell 75: 324–339.e11 31155380 10.1016/j.molcel.2019.05.008 PMC6675935

[embj2023114378-bib-0059] Hogg JR , Goff SP (2010) Upf1 senses 3′UTR length to potentiate mRNA decay. Cell 143: 379–389 21029861 10.1016/j.cell.2010.10.005 PMC2981159

[embj2023114378-bib-0060] Holbrook JA , Neu‐Yilik G , Hentze MW , Kulozik AE (2004) Nonsense‐mediated decay approaches the clinic. Nat Genet 36: 801–808 15284851 10.1038/ng1403

[embj2023114378-bib-0061] van Hoof A , Frischmeyer PA , Dietz HC , Parker R (2002) Exosome‐mediated recognition and degradation of mRNAs lacking a termination codon. Science 295: 2262–2264 11910110 10.1126/science.1067272

[embj2023114378-bib-0062] Huang L , Lou C‐H , Chan W , Shum EY , Shao A , Stone E , Karam R , Song H‐W , Wilkinson MF (2011) RNA homeostasis governed by cell type‐specific and branched feedback loops acting on NMD. Mol Cell 43: 950–961 21925383 10.1016/j.molcel.2011.06.031 PMC4281029

[embj2023114378-bib-0063] Hug N , Cáceres JF (2014) The RNA helicase DHX34 activates NMD by promoting a transition from the surveillance to the decay‐inducing complex. Cell Rep 8: 1845–1856 25220460 10.1016/j.celrep.2014.08.020 PMC4534575

[embj2023114378-bib-0064] Hug N , Longman D , Cáceres JF (2016) Mechanism and regulation of the nonsense‐mediated decay pathway. Nucleic Acids Res 44: 1483–1495 26773057 10.1093/nar/gkw010 PMC4770240

[embj2023114378-bib-0065] Hug N , Aitken S , Longman D , Raab M , Armes H , Mann AR , Rio‐Machin A , Fitzgibbon J , Rouault‐Pierre K , Caceres JF (2022) A dual role for the RNA helicase DHX34 in NMD and pre‐mRNA splicing and its function in hematopoietic differentiation. RNA 28: 1224–1238 35768279 10.1261/rna.079277.122 PMC9380745

[embj2023114378-bib-0066] Huntzinger E , Kashima I , Fauser M , Saulière J , Izaurralde E (2008) SMG6 is the catalytic endonuclease that cleaves mRNAs containing nonsense codons in metazoan. RNA 14: 2609–2617 18974281 10.1261/rna.1386208 PMC2590965

[embj2023114378-bib-0067] Hurt JA , Robertson AD , Burge CB (2013) Global analyses of UPF1 binding and function reveal expanded scope of nonsense‐mediated mRNA decay. Genome Res 23: 1636–1650 23766421 10.1101/gr.157354.113 PMC3787261

[embj2023114378-bib-0068] Huth M , Santini L , Galimberti E , Ramesmayer J , Titz‐Teixeira F , Sehlke R , Oberhuemer M , Stummer S , Herzog V , Garmhausen M *et al* (2022) NMD is required for timely cell fate transitions by fine‐tuning gene expression and regulating translation. Genes Dev 36: 348–367 35241478 10.1101/gad.347690.120 PMC8973849

[embj2023114378-bib-0069] Ikeuchi K , Tesina P , Matsuo Y , Sugiyama T , Cheng J , Saeki Y , Tanaka K , Becker T , Beckmann R , Inada T (2019) Collided ribosomes form a unique structural interface to induce Hel2‐driven quality control pathways. EMBO J 38: e100276 30609991 10.15252/embj.2018100276 PMC6396155

[embj2023114378-bib-0070] Inglis AJ , Guna A , Gálvez‐Merchán Á , Pal A , Esantsi TK , Keys HR , Frenkel EM , Oania R , Weissman JS , Voorhees RM (2023) Coupled protein quality control during nonsense‐mediated mRNA decay. J. Cell Sci 136: jcs261216 37218462 10.1242/jcs.261216 PMC10234110

[embj2023114378-bib-0071] Isken O , Kim YK , Hosoda N , Mayeur GL , Hershey JWB , Maquat LE (2008) Upf1 phosphorylation triggers translational repression during nonsense‐mediated mRNA decay. Cell 133: 314–327 18423202 10.1016/j.cell.2008.02.030 PMC4193665

[embj2023114378-bib-0072] Ito‐Harashima S , Kuroha K , Tatematsu T , Inada T (2007) Translation of the poly(A) tail plays crucial roles in nonstop mRNA surveillance via translation repression and protein destabilization by proteasome in yeast. Genes Dev 21: 519–524 17344413 10.1101/gad.1490207 PMC1820893

[embj2023114378-bib-0073] Ivanov PV , Gehring NH , Kunz JB , Hentze MW , Kulozik AE (2008) Interactions between UPF1, eRFs, PABP and the exon junction complex suggest an integrated model for mammalian NMD pathways. EMBO J 27: 736–747 18256688 10.1038/emboj.2008.17 PMC2265754

[embj2023114378-bib-0074] Izumi N , Yamashita A , Iwamatsu A , Kurata R , Nakamura H , Saari B , Hirano H , Anderson P , Ohno S (2010) AAA+ proteins RUVBL1 and RUVBL2 coordinate PIKK activity and function in nonsense‐mediated mRNA decay. Sci Signal 3: ra27 20371770 10.1126/scisignal.2000468

[embj2023114378-bib-0075] Jackson KL , Dayton RD , Orchard EA , Ju S , Ringe D , Petsko GA , Maquat LE , Klein RL (2015) Preservation of forelimb function by UPF1 gene therapy in a rat model of TDP‐43‐induced motor paralysis. Gene Ther 22: 20–28 25354681 10.1038/gt.2014.101 PMC4924570

[embj2023114378-bib-0076] Jaffrey SR , Wilkinson MF (2018) Nonsense‐mediated RNA decay in the brain: emerging modulator of neural development and disease. Nat Rev Neurosci 19: 715–728 30410025 10.1038/s41583-018-0079-z PMC6396682

[embj2023114378-bib-0077] Jolly LA , Homan CC , Jacob R , Barry S , Gecz J (2013) The UPF3B gene, implicated in intellectual disability, autism, ADHD and childhood onset schizophrenia regulates neural progenitor cell behaviour and neuronal outgrowth. Hum Mol Genet 22: 4673–4687 23821644 10.1093/hmg/ddt315

[embj2023114378-bib-0078] Juszkiewicz S , Hegde RS (2017) Initiation of quality control during poly(A) translation requires site‐specific ribosome ubiquitination. Mol Cell 65: 743–750.e4 28065601 10.1016/j.molcel.2016.11.039 PMC5316413

[embj2023114378-bib-0079] Juszkiewicz S , Chandrasekaran V , Lin Z , Kraatz S , Ramakrishnan V , Hegde RS (2018) ZNF598 is a quality control sensor of collided ribosomes. Mol Cell 72: 469–481.e7 30293783 10.1016/j.molcel.2018.08.037 PMC6224477

[embj2023114378-bib-0080] Juszkiewicz S , Speldewinde SH , Wan L , Svejstrup JQ , Hegde RS (2020) The ASC‐1 complex disassembles collided ribosomes. Mol Cell 79: 603–614.e8 32579943 10.1016/j.molcel.2020.06.006 PMC7447978

[embj2023114378-bib-0081] Karam R , Lou C‐HC‐H , Kroeger H , Huang L , Lin JH , Wilkinson MF (2015) The unfolded protein response is shaped by the NMD pathway. EMBO Rep 16: 599–609 25807986 10.15252/embr.201439696 PMC4428047

[embj2023114378-bib-0082] Karousis ED , Mühlemann O (2022) The broader sense of nonsense. Trends Biochem Sci 47: 921–935 35780009 10.1016/j.tibs.2022.06.003

[embj2023114378-bib-0083] Karousis ED , Gurzeler L‐A , Annibaldis G , Dreos R , Mühlemann O (2020) Human NMD ensues independently of stable ribosome stalling. Nat Commun 11: 4134 32807779 10.1038/s41467-020-17974-z PMC7431590

[embj2023114378-bib-0084] Karousis ED , Gypas F , Zavolan M , Mühlemann O (2021) Nanopore sequencing reveals endogenous NMD‐targeted isoforms in human cells. Genome Biol 22: 223 34389041 10.1186/s13059-021-02439-3 PMC8361881

[embj2023114378-bib-0085] Kashima I , Yamashita A , Izumi N , Kataoka N , Morishita R , Hoshino S , Ohno M , Dreyfuss G , Ohno S (2006) Binding of a novel SMG‐1‐Upf1‐eRF1‐eRF3 complex (SURF) to the exon junction complex triggers Upf1 phosphorylation and nonsense‐mediated mRNA decay. Genes Dev 20: 355–367 16452507 10.1101/gad.1389006 PMC1361706

[embj2023114378-bib-0086] Katsioudi G , Dreos R , Arpa ES , Gaspari S , Liechti A , Sato M , Gabriel CH , Kramer A , Brown SA , Gatfield D (2023) A conditional Smg6 mutant mouse model reveals circadian clock regulation through the nonsense‐mediated mRNA decay pathway. Sci. Adv 9: eade2828 36638184 10.1126/sciadv.ade2828 PMC9839329

[embj2023114378-bib-0087] Kaygun H , Marzluff WF (2005) Regulated degradation of replication‐dependent histone mRNAs requires both ATR and Upf1. Nat Struct Mol Biol 12: 794–800 16086026 10.1038/nsmb972

[embj2023114378-bib-0088] Kervestin S , Jacobson A (2012) NMD: a multifaceted response to premature translational termination. Nat Rev Mol Cell Biol 13: 700–712 23072888 10.1038/nrm3454 PMC3970730

[embj2023114378-bib-0089] Kim YK , Maquat LE (2019) UPFront and center in RNA decay: UPF1 in nonsense‐mediated mRNA decay and beyond. RNA 25: 407–422 30655309 10.1261/rna.070136.118 PMC6426291

[embj2023114378-bib-0090] Kim YK , Furic L , Desgroseillers L , Maquat LE (2005) Mammalian Staufen1 recruits Upf1 to specific mRNA 3′UTRs so as to elicit mRNA decay. Cell 120: 195–208 15680326 10.1016/j.cell.2004.11.050

[embj2023114378-bib-0091] Kim YJ , Nomakuchi T , Papaleonidopoulou F , Yang L , Zhang Q , Krainer AR (2022) Gene‐specific nonsense‐mediated mRNA decay targeting for cystic fibrosis therapy. Nat Commun 13: 2978 35624092 10.1038/s41467-022-30668-y PMC9142507

[embj2023114378-bib-0092] Koutmou KS , Schuller AP , Brunelle JL , Radhakrishnan A , Djuranovic S , Green R (2015) Ribosomes slide on lysine‐encoding homopolymeric A stretches. Elife 4: e05534 25695637 10.7554/eLife.05534 PMC4363877

[embj2023114378-bib-0093] Kuroha K , Ando K , Nakagawa R , Inada T (2013) The Upf factor complex interacts with aberrant products derived from mRNAs containing a premature termination codon and facilitates their proteasomal degradation. J Biol Chem 288: 28630–28640 23928302 10.1074/jbc.M113.460691 PMC3789962

[embj2023114378-bib-0094] Kurosaki T , Li W , Hoque M , Popp MW , Ermolenko DN , Tian B , Maquat LE (2014) A post‐translational regulatory switch on UPF1 controls targeted mRNA degradation. Genes Dev 28: 1900–1916 25184677 10.1101/gad.245506.114 PMC4197951

[embj2023114378-bib-0095] Kurosaki T , Miyoshi K , Myers JR , Maquat LE (2018) NMD‐degradome sequencing reveals ribosome‐bound intermediates with 3′‐end non‐templated nucleotides. Nat Struct Mol Biol 25: 940–950 30275517 10.1038/s41594-018-0132-7 PMC8262411

[embj2023114378-bib-0096] Kurosaki T , Popp MW , Maquat LE (2019) Quality and quantity control of gene expression by nonsense‐mediated mRNA decay. Nat Rev Mol Cell Biol 20: 406–420 30992545 10.1038/s41580-019-0126-2 PMC6855384

[embj2023114378-bib-0097] Kurosaki T , Imamachi N , Pröschel C , Mitsutomi S , Nagao R , Akimitsu N , Maquat LE (2021) Loss of the fragile X syndrome protein FMRP results in misregulation of nonsense‐mediated mRNA decay. Nat Cell Biol 23: 40–48 33420492 10.1038/s41556-020-00618-1 PMC8273690

[embj2023114378-bib-0098] Langer LM , Bonneau F , Gat Y , Conti E (2021) Cryo‐EM reconstructions of inhibitor‐bound SMG1 kinase reveal an autoinhibitory state dependent on SMG8. Elife 10: e72353 34698635 10.7554/eLife.72353 PMC8592573

[embj2023114378-bib-0099] Le Hir H , Izaurralde E , Maquat LE , Moore MJ (2000a) The spliceosome deposits multiple proteins 20–24 nucleotides upstream of mRNA exon‐exon junctions. EMBO J 19: 6860–6869 11118221 10.1093/emboj/19.24.6860 PMC305905

[embj2023114378-bib-0100] Le Hir H , Moore MJ , Maquat LE (2000b) Pre‐mRNA splicing alters mRNP composition: evidence for stable association of proteins at exon‐exon junctions. Genes Dev 14: 1098–1108 10809668 PMC316578

[embj2023114378-bib-0101] Lee SR , Pratt GA , Martinez FJ , Yeo GW , Lykke‐Andersen J (2015) Target discrimination in nonsense‐mediated mRNA decay requires Upf1 ATPase activity. Mol. Cell 59: 413–425 26253027 10.1016/j.molcel.2015.06.036 PMC4673969

[embj2023114378-bib-0102] Lindeboom RGH , Supek F , Lehner B (2016) The rules and impact of nonsense‐mediated mRNA decay in human cancers. Nat Genet 48: 1112–1118 27618451 10.1038/ng.3664 PMC5045715

[embj2023114378-bib-0103] Loh B , Jonas S , Izaurralde E (2013) The SMG5‐SMG7 heterodimer directly recruits the CCR4‐NOT deadenylase complex to mRNAs containing nonsense codons via interaction with POP2. Genes Dev 27: 2125–2138 24115769 10.1101/gad.226951.113 PMC3850096

[embj2023114378-bib-0104] Longman D , Plasterk RHA , Johnstone IL , Cáceres JF (2007) Mechanistic insights and identification of two novel factors in the *C. elegans* NMD pathway. Genes Dev 21: 1075–1085 17437990 10.1101/gad.417707 PMC1855233

[embj2023114378-bib-0105] Longman D , Hug N , Keith M , Anastasaki C , Patton EE , Grimes G , Cáceres JF (2013) DHX34 and NBAS form part of an autoregulatory NMD circuit that regulates endogenous RNA targets in human cells, zebrafish and *Caenorhabditis elegans* . Nucleic Acids Res 41: 8319–8331 23828042 10.1093/nar/gkt585 PMC3783168

[embj2023114378-bib-0106] Longman D , Jackson‐Jones KA , Maslon MM , Murphy LC , Young RS , Stoddart JJ , Hug N , Taylor MS , Papadopoulos DK , Cáceres JF (2020) Identification of a localized nonsense‐mediated decay pathway at the endoplasmic reticulum. Genes Dev 34: 1075–1088 32616520 10.1101/gad.338061.120 PMC7397857

[embj2023114378-bib-0107] López‐Perrote A , Castaño R , Melero R , Zamarro T , Kurosawa H , Ohnishi T , Uchiyama A , Aoyagi K , Buchwald G , Kataoka N *et al* (2016) Human nonsense‐mediated mRNA decay factor UPF2 interacts directly with eRF3 and the SURF complex. Nucleic Acids Res 44: 1909–1923 26740584 10.1093/nar/gkv1527 PMC4770235

[embj2023114378-bib-0108] López‐Perrote A , Hug N , González‐Corpas A , Rodríguez CF , Serna M , García‐Martín C , Boskovic J , Fernandez‐Leiro R , Caceres JF , Llorca O (2020) Regulation of RUVBL1‐RUVBL2 AAA‐ATPases by the nonsense‐mediated mRNA decay factor DHX34, as evidenced by Cryo‐EM. Elife 9: e63042 33205750 10.7554/eLife.63042 PMC7707835

[embj2023114378-bib-0109] Matsuo Y , Ikeuchi K , Saeki Y , Iwasaki S , Schmidt C , Udagawa T , Sato F , Tsuchiya H , Becker T , Tanaka K *et al* (2017) Ubiquitination of stalled ribosome triggers ribosome‐associated quality control. Nat. Commun 8: 159 28757607 10.1038/s41467-017-00188-1 PMC5534433

[embj2023114378-bib-0110] Matsuo Y , Tesina P , Nakajima S , Mizuno M , Endo A , Buschauer R , Cheng J , Shounai O , Ikeuchi K , Saeki Y *et al* (2020) RQT complex dissociates ribosomes collided on endogenous RQC substrate SDD1. Nat Struct Mol Biol 27: 323–332 32203490 10.1038/s41594-020-0393-9

[embj2023114378-bib-0111] Melero R , Hug N , Lopez‐Perrote A , Yamashita A , Caceres JF , Llorca O (2016) The RNA helicase DHX34 functions as a scaffold for SMG1‐mediated UPF1 phosphorylation. Nat. Commun 7: 10585 26841701 10.1038/ncomms10585 PMC4743010

[embj2023114378-bib-0112] Metze S , Herzog VA , Ruepp M‐D , Mühlemann O (2013) Comparison of EJC‐enhanced and EJC‐independent NMD in human cells reveals two partially redundant degradation pathways. RNA 19: 1432–1448 23962664 10.1261/rna.038893.113 PMC3854533

[embj2023114378-bib-0113] Monem PC , Vidyasagar N , Piatt AL , Sehgal E , Arribere JA (2023) Ubiquitination of stalled ribosomes enables mRNA decay via HBS‐1 and NONU‐1 in vivo. PLoS Genet 19: e1010577 36626369 10.1371/journal.pgen.1010577 PMC9870110

[embj2023114378-bib-0114] Mort M , Ivanov D , Cooper DN , Chuzhanova NA (2008) A meta‐analysis of nonsense mutations causing human genetic disease. Hum Mutat 29: 1037–1047 18454449 10.1002/humu.20763

[embj2023114378-bib-0115] Nagy E , Maquat LE (1998) A rule for termination‐codon position within intron‐containing genes: when nonsense affects RNA abundance. Trends Biochem Sci 23: 198–199 9644970 10.1016/s0968-0004(98)01208-0

[embj2023114378-bib-0116] Neu‐Yilik G , Raimondeau E , Eliseev B , Yeramala L , Amthor B , Deniaud A , Huard K , Kerschgens K , Hentze MW , Schaffitzel C *et al* (2017) Dual function of UPF3B in early and late translation termination. EMBO J 36: 2968–2986 28899899 10.15252/embj.201797079 PMC5641913

[embj2023114378-bib-0117] Nguyen LS , Kim H‐G , Rosenfeld JA , Shen Y , Gusella JF , Lacassie Y , Layman LC , Shaffer LG , Gécz J (2013) Contribution of copy number variants involving nonsense‐mediated mRNA decay pathway genes to neuro‐developmental disorders. Hum Mol Genet 22: 1816–1825 23376982 10.1093/hmg/ddt035

[embj2023114378-bib-0118] Nguyen LS , Wilkinson MF , Gecz J (2014) Nonsense‐mediated mRNA decay: inter‐individual variability and human disease. Neurosci Biobehav Rev 46: 175–186 24239855 10.1016/j.neubiorev.2013.10.016 PMC4021004

[embj2023114378-bib-0119] Nogueira G , Fernandes R , García‐Moreno JF , Romão L (2021) Nonsense‐mediated RNA decay and its bipolar function in cancer. Mol Cancer 20: 72 33926465 10.1186/s12943-021-01364-0 PMC8082775

[embj2023114378-bib-0120] Ortega JA , Daley EL , Kour S , Samani M , Tellez L , Smith HS , Hall EA , Esengul YT , Tsai Y‐H , Gendron TF *et al* (2020) Nucleocytoplasmic proteomic analysis uncovers eRF1 and nonsense‐mediated decay as modifiers of ALS/FTD C9orf72 toxicity. Neuron 106: 90–107.e13 32059759 10.1016/j.neuron.2020.01.020 PMC7272217

[embj2023114378-bib-0121] Palma M , Leroy C , Salomé‐Desnoulez S , Werkmeister E , Kong R , Mongy M , Le Hir H , Lejeune F (2021) A role for AKT1 in nonsense‐mediated mRNA decay. Nucleic Acids Res 49: 11022–11037 34634811 10.1093/nar/gkab882 PMC8565340

[embj2023114378-bib-0122] Park E , Gleghorn ML , Maquat LE (2013) Staufen2 functions in Staufen1‐mediated mRNA decay by binding to itself and its paralog and promoting UPF1 helicase but not ATPase activity. Proc Natl Acad Sci USA 110: 405–412 23263869 10.1073/pnas.1213508110 PMC3545820

[embj2023114378-bib-0123] Pastor F , Kolonias D , Giangrande PH , Gilboa E (2010) Induction of tumour immunity by targeted inhibition of nonsense‐mediated mRNA decay. Nature 465: 227–230 20463739 10.1038/nature08999 PMC3107067

[embj2023114378-bib-0124] Pisareva VP , Skabkin MA , Hellen CUT , Pestova TV , Pisarev AV (2011) Dissociation by Pelota, Hbs1 and ABCE1 of mammalian vacant 80S ribosomes and stalled elongation complexes. EMBO J 30: 1804–1817 21448132 10.1038/emboj.2011.93 PMC3101999

[embj2023114378-bib-0125] Popp MW , Maquat LE (2015) Attenuation of nonsense‐mediated mRNA decay facilitates the response to chemotherapeutics. Nat Commun 6: 6632 25808464 10.1038/ncomms7632 PMC4375787

[embj2023114378-bib-0126] Powers KT , Szeto J‐YA , Schaffitzel C (2020) New insights into no‐go, non‐stop and nonsense‐mediated mRNA decay complexes. Curr Opin Struct Biol 65: 110–118 32688260 10.1016/j.sbi.2020.06.011

[embj2023114378-bib-0127] Rio‐Machin A , Vulliamy T , Hug N , Walne A , Tawana K , Cardoso S , Ellison A , Pontikos N , Wang J , Tummala H *et al* (2020) The complex genetic landscape of familial MDS and AML reveals pathogenic germline variants. Nat Commun 11: 1044 32098966 10.1038/s41467-020-14829-5 PMC7042299

[embj2023114378-bib-0128] Rivas MA , Pirinen M , Conrad DF , Lek M , Tsang EK , Karczewski KJ , Maller JB , Kukurba KR , DeLuca DS , Fromer M *et al* (2015) Effect of predicted protein‐truncating genetic variants on the human transcriptome. Science 348: 666–669 25954003 10.1126/science.1261877 PMC4537935

[embj2023114378-bib-0129] Saito S , Hosoda N , Hoshino SI (2013) The Hbs1‐Dom34 protein complex functions in non‐stop mRNA decay in mammalian cells. J Biol Chem 288: 17832–17843 23667253 10.1074/jbc.M112.448977 PMC3682582

[embj2023114378-bib-0130] Sato H , Singer RH (2021) Cellular variability of nonsense‐mediated mRNA decay. Nat Commun 12: 7203 34893608 10.1038/s41467-021-27423-0 PMC8664836

[embj2023114378-bib-0131] Serin G , Gersappe A , Black JD , Aronoff R , Maquat LE (2001) Identification and characterization of human orthologues to Saccharomyces cerevisiae Upf2 protein and Upf3 protein (*Caenorhabditis elegans* SMG‐4). Mol Cell Biol 21: 209–223 11113196 10.1128/MCB.21.1.209-223.2001 PMC88795

[embj2023114378-bib-0132] Shaheen R , Anazi S , Ben‐Omran T , Seidahmed MZ , Caddle LB , Palmer K , Ali R , Alshidi T , Hagos S , Goodwin L *et al* (2016) Mutations in SMG9, encoding an essential component of nonsense‐mediated decay machinery, cause a multiple congenital anomaly syndrome in humans and mice. Am J Hum Genet 98: 643–652 27018474 10.1016/j.ajhg.2016.02.010 PMC4833216

[embj2023114378-bib-0133] Shao S , von der Malsburg K , Hegde RS (2013) Listerin‐dependent nascent protein ubiquitination relies on ribosome subunit dissociation. Mol Cell 50: 637–648 23685075 10.1016/j.molcel.2013.04.015 PMC3719020

[embj2023114378-bib-0134] Shoemaker CJ , Green R (2012) Translation drives mRNA quality control. Nat Struct Mol Biol 19: 594–601 22664987 10.1038/nsmb.2301 PMC4299859

[embj2023114378-bib-0135] Shoemaker CJ , Eyler DE , Green R (2010) Dom34:Hbs1 promotes subunit dissociation and peptidyl‐tRNA drop‐off to initiate no‐go decay. Science 330: 369–372 20947765 10.1126/science.1192430 PMC4022135

[embj2023114378-bib-0136] Sieber J , Hauer C , Bhuvanagiri M , Leicht S , Krijgsveld J , Neu‐Yilik G , Hentze MW , Kulozik AE (2016) Proteomic analysis reveals branch‐specific regulation of the unfolded protein response by nonsense‐mediated mRNA decay. Mol Cell Proteomics 15: 1584–1597 26896796 10.1074/mcp.M115.054056 PMC4858941

[embj2023114378-bib-0137] Simms CL , Thomas EN , Zaher HS (2016) Ribosome‐based quality control of mRNA and nascent peptides. Wiley Interdiscip Rev RNA 8: 10.1002/wrna.1366 PMC5116004 27193249

[embj2023114378-bib-0138] Sinha NK , Ordureau A , Best KM , Saba JA , Zinshteyn B , Sundaramoorthy E , Fulzele A , Garshott DM , Denk T , Thoms M *et al* (2020) EDF1 coordinates cellular responses to ribosome collisions. Elife 9: 1–84 10.7554/eLife.58828 PMC7486125 32744497

[embj2023114378-bib-0139] Staufner C , Peters B , Wagner M , Alameer S , Barić I , Broué P , Bulut D , Church JA , Crushell E , Dalgıç B *et al* (2020) Defining clinical subgroups and genotype‐phenotype correlations in NBAS‐associated disease across 110 patients. Genet Med 22: 610–621 31761904 10.1038/s41436-019-0698-4

[embj2023114378-bib-0140] Steckelberg A‐L , Boehm V , Gromadzka AM , Gehring NH (2012) CWC22 connects pre‐mRNA splicing and exon junction complex assembly. Cell Rep 2: 454–461 22959432 10.1016/j.celrep.2012.08.017

[embj2023114378-bib-0141] Sun X , Perlick HA , Dietz HC , Maquat LE (1998) A mutated human homologue to yeast Upf1 protein has a dominant‐negative effect on the decay of nonsense‐containing mRNAs in mammalian cells. Proc Natl Acad Sci USA 95: 10009–10014 9707591 10.1073/pnas.95.17.10009 PMC21452

[embj2023114378-bib-0142] Sun Y , Eshov A , Zhou J , Isiktas AU , Guo JU (2020) C9orf72 arginine‐rich dipeptide repeats inhibit UPF1‐mediated RNA decay via translational repression. Nat Commun 11: 3354 32620797 10.1038/s41467-020-17129-0 PMC7335171

[embj2023114378-bib-0143] Takahashi S , Araki Y , Ohya Y , Sakuno T , Hoshino S , Kontani K , Nishina H , Katada T (2008) Upf1 potentially serves as a RING‐related E3 ubiquitin ligase via its association with Upf3 in yeast. RNA 14: 1950–1958 18676617 10.1261/rna.536308 PMC2525956

[embj2023114378-bib-0144] Tan K , Stupack DG , Wilkinson MF (2022) Nonsense‐mediated RNA decay: an emerging modulator of malignancy. Nat Rev Cancer 22: 437–451 35624152 10.1038/s41568-022-00481-2 PMC11009036

[embj2023114378-bib-0145] Terrey M , Adamson SI , Chuang JH , Ackerman SL (2021) Defects in translation‐dependent quality control pathways lead to convergent molecular and neurodevelopmental pathology. Elife 10: e66904 33899734 10.7554/eLife.66904 PMC8075583

[embj2023114378-bib-0146] Tesina P , Lessen LN , Buschauer R , Cheng J , Wu CC , Berninghausen O , Buskirk AR , Becker T , Beckmann R , Green R (2020) Molecular mechanism of translational stalling by inhibitory codon combinations and poly(A) tracts. EMBO J 39: e103365 31858614 10.15252/embj.2019103365 PMC6996574

[embj2023114378-bib-0147] Thermann R , Neu‐Yilik G , Deters A , Frede U , Wehr K , Hagemeier C , Hentze MW , Kulozik AE (1998) Binary specification of nonsense codons by splicing and cytoplasmic translation. EMBO J 17: 3484–3494 9628884 10.1093/emboj/17.12.3484 PMC1170685

[embj2023114378-bib-0148] Trcek T , Sato H , Singer RH , Maquat LE (2013) Temporal and spatial characterization of nonsense‐mediated mRNA decay. Genes Dev 27: 541–551 23431032 10.1101/gad.209635.112 PMC3605467

[embj2023114378-bib-0149] Tsuboi T , Kuroha K , Kudo K , Makino S , Inoue E , Kashima I , Inada T (2012) Dom34: Hbs1 plays a general role in quality‐control systems by dissociation of a stalled ribosome at the 3′ end of aberrant mRNA. Mol Cell 46: 518–529 22503425 10.1016/j.molcel.2012.03.013

[embj2023114378-bib-0150] Tuck AC , Rankova A , Arpat AB , Liechti LA , Hess D , Iesmantavicius V , Castelo‐Szekely V , Gatfield D , Bühler M (2020) Mammalian RNA decay pathways are highly specialized and widely linked to translation. Mol Cell 77: 1222–1236.e13 32048998 10.1016/j.molcel.2020.01.007 PMC7083229

[embj2023114378-bib-0151] Unterholzner L , Izaurralde E (2004) SMG7 acts as a molecular link between mRNA surveillance and mRNA decay. Mol Cell 16: 587–596 15546618 10.1016/j.molcel.2004.10.013

[embj2023114378-bib-0152] Veltri AJ , D’Orazio KN , Lessen LN , Loll‐Krippleber R , Brown GW , Green R (2022) Distinct elongation stalls during translation are linked with distinct pathways for mRNA degradation. Elife 11: e76038 35894211 10.7554/eLife.76038 PMC9352352

[embj2023114378-bib-0153] Wallefeld W , Krause S , Nowak KJ , Dye D , Horváth R , Molnár Z , Szabó M , Hashimoto K , Reina C , De Carlos J *et al* (2006) Severe nemaline myopathy caused by mutations of the stop codon of the skeletal muscle alpha actin gene (ACTA1). Neuromuscul Disord 16: 541–547 16945536 10.1016/j.nmd.2006.07.018

[embj2023114378-bib-0154] Wallmeroth D , Lackmann J , Kueckelmann S , Altmüller J , Dieterich C , Boehm V , Gehring NH (2022) Human UPF3A and UPF3B enable fault‐tolerant activation of nonsense‐mediated mRNA decay. EMBO J 41: e109191 35451084 10.15252/embj.2021109191 PMC9108619

[embj2023114378-bib-0155] Walter P , Ron D (2011) The unfolded protein response: from stress pathway to homeostatic regulation. Science 334: 1081–1086 22116877 10.1126/science.1209038

[embj2023114378-bib-0156] Wang D , Zavadil J , Martin L , Parisi F , Friedman E , Levy D , Harding H , Ron D , Gardner LB (2011) Inhibition of nonsense‐mediated RNA decay by the tumor microenvironment promotes tumorigenesis. Mol Cell Biol 31: 3670–3680 21730287 10.1128/MCB.05704-11 PMC3165546

[embj2023114378-bib-0157] Wu Q , Bazzini AA (2023) Translation and mRNA stability control. Annu Rev Biochem 92: 227–245 37001134 10.1146/annurev-biochem-052621-091808

[embj2023114378-bib-0158] Wu B , Eliscovich C , Yoon YJ , Singer RH (2016) Translation dynamics of single mRNAs in live cells and neurons. Science 352: 1430–1435 27313041 10.1126/science.aaf1084 PMC4939616

[embj2023114378-bib-0159] Wu Q , Medina SG , Kushawah G , Devore ML , Castellano LA , Hand JM , Wright M , Bazzini AA (2019) Translation affects mRNA stability in a codon‐dependent manner in human cells. Elife 8: e45396 31012849 10.7554/eLife.45396 PMC6529216

[embj2023114378-bib-0160] Xu W , Bao P , Jiang X , Wang H , Qin M , Wang R , Wang T , Yang Y , Lorenzini I , Liao L *et al* (2019) Reactivation of nonsense‐mediated mRNA decay protects against C9orf72 dipeptide‐repeat neurotoxicity. Brain 142: 1349–1364 30938419 10.1093/brain/awz070 PMC6487333

[embj2023114378-bib-0161] Yamashita A , Ohnishi T , Kashima I , Taya Y , Ohno S (2001) Human SMG‐1, a novel phosphatidylinositol 3‐kinase‐related protein kinase, associates with components of the mRNA surveillance complex and is involved in the regulation of nonsense‐mediated mRNA decay. Genes Dev 15: 2215–2228 11544179 10.1101/gad.913001 PMC312771

[embj2023114378-bib-0162] Yamashita A , Izumi N , Kashima I , Ohnishi T , Saari B , Katsuhata Y , Muramatsu R , Morita T , Iwamatsu A , Hachiya T *et al* (2009) SMG‐8 and SMG‐9, two novel subunits of the SMG‐1 complex, regulate remodeling of the mRNA surveillance complex during nonsense‐mediated mRNA decay. Genes Dev 23: 1091–1105 19417104 10.1101/gad.1767209 PMC2682953

[embj2023114378-bib-0163] Yepiskoposyan H , Aeschimann F , Nilsson D , Okoniewski M , Mühlemann O (2011) Autoregulation of the nonsense‐mediated mRNA decay pathway in human cells. RNA 17: 2108–2118 22028362 10.1261/rna.030247.111 PMC3222124

[embj2023114378-bib-0164] Yi Z , Arvola RM , Myers S , Dilsavor CN , Abu Alhasan R , Carter BN , Patton RD , Bundschuh R , Singh G (2022) Mammalian UPF3A and UPF3B can activate nonsense‐mediated mRNA decay independently of their exon junction complex binding. EMBO J 41: e109202 35451102 10.15252/embj.2021109202 PMC9108626

[embj2023114378-bib-0165] Zetoune AB , Fontanière S , Magnin D , Anczuków O , Buisson M , Zhang CX , Mazoyer S (2008) Comparison of nonsense‐mediated mRNA decay efficiency in various murine tissues. BMC Genet 9: 83 19061508 10.1186/1471-2156-9-83 PMC2607305

[embj2023114378-bib-0166] Zhu X , Zhang H , Mendell JT (2020) Ribosome recycling by ABCE1 links lysosomal function and iron homeostasis to 3′ UTR‐directed regulation and nonsense‐mediated decay. Cell Rep 32: 107895 32668236 10.1016/j.celrep.2020.107895 PMC7433747

[embj2023114378-bib-0167] Zinshteyn B , Sinha NK , Enam SU , Koleske B , Green R (2021) Translational repression of NMD targets by GIGYF2 and EIF4E2. PLoS Genet 17: e1009813 34665823 10.1371/journal.pgen.1009813 PMC8555832

[embj2023114378-bib-0168] Zünd D , Gruber AR , Zavolan M , Mühlemann O (2013) Translation‐dependent displacement of UPF1 from coding sequences causes its enrichment in 3′ UTRs. Nat Struct Mol Biol 20: 936–943 23832275 10.1038/nsmb.2635

